# Stability and Competition in Multi-spike Models of Spike-Timing Dependent Plasticity

**DOI:** 10.1371/journal.pcbi.1004750

**Published:** 2016-03-03

**Authors:** Baktash Babadi, L. F. Abbott

**Affiliations:** 1 Center for Theoretical Neuroscience, Department of Neuroscience, Columbia University, New York, New York, United States of America; 2 Swartz Program in Theoretical Neuroscience, Center for Brain Science, Harvard University, Cambridge, Massachusetts, United States of America; École Normale Supérieure, College de France, CNRS, FRANCE

## Abstract

Spike-timing dependent plasticity (STDP) is a widespread plasticity mechanism in the nervous system. The simplest description of STDP only takes into account pairs of pre- and postsynaptic spikes, with potentiation of the synapse when a presynaptic spike precedes a postsynaptic spike and depression otherwise. In light of experiments that explored a variety of spike patterns, the pair-based STDP model has been augmented to account for multiple pre- and postsynaptic spike interactions. As a result, a number of different “multi-spike” STDP models have been proposed based on different experimental observations. The behavior of these models at the population level is crucial for understanding mechanisms of learning and memory. The challenging balance between the stability of a population of synapses and their competitive modification is well studied for pair-based models, but it has not yet been fully analyzed for multi-spike models. Here, we address this issue through numerical simulations of an integrate-and-fire model neuron with excitatory synapses subject to STDP described by three different proposed multi-spike models. We also analytically calculate average synaptic changes and fluctuations about these averages. Our results indicate that the different multi-spike models behave quite differently at the population level. Although each model can produce synaptic competition in certain parameter regions, none of them induces synaptic competition with its originally fitted parameters. The dichotomy between synaptic stability and Hebbian competition, which is well characterized for pair-based STDP models, persists in multi-spike models. However, anti-Hebbian competition can coexist with synaptic stability in some models. We propose that the collective behavior of synaptic plasticity models at the population level should be used as an additional guideline in applying phenomenological models based on observations of single synapses.

## Author Summary

Synaptic plasticity is believed to underlie learning and memory by competitive strengthening and weakening of synapses in neural networks. However, the ability to form new memories while maintaining the old ones involves an intricate balance between synaptic stability and competition. In one of the most widespread such mechanisms, spike-timing dependent plasticity (STDP), the temporal order of pre- and postsynaptic spiking across a synapse determines whether it is strengthened or weakened. Early description of STDP only took into account pairs of pre- and postsynaptic spikes. However, more recent experimental results showed that the “pair-based” description is not sufficient to fully account for synaptic modifications under STDP, and motivated more complex “multi-spike” STDP models. While the conditions under which the pair-based STDP leads to synaptic stability and/or competition are well studied, it is not clear when and how multi-spike STDP models lead to synaptic stability and competition. Here, we address these questions through numerical simulation and analysis of a population of plastic excitatory synapses that converge to a neuron. We show that different multi-spike STDP models can induce synaptic stability and competition under radically different conditions, which have important implications in relating learning and memory to biophysical properties of synapses.

## Introduction

Spike-timing dependent plasticity (STDP) is a form of activity-dependent synaptic plasticity that appears throughout the nervous system [1, 2, 3]. In STDP, pairs of pre- and postsynaptic action potentials potentiate a synapse when the presynaptic spike precedes the postsynaptic spike, and depress it for the reverse order [4, 5]. However, when multiple pre- and postsynaptic spikes occur across a synapse over a short interval of time, the resulting plasticity depends on their timing in a more complex manner. For example, pair-based STDP models predict that “pre-post-pre” and “post-pre-post” triplets of spikes with the same pairwise intervals should induce the same plasticity, but experiments indicate that these two triplet patterns have different effects [6, 7]. This and similar contradictions motivated the development of multi-spike models of STDP, which go beyond pairwise interactions of pre- and postsynaptic spikes (see [8] for a review). Here, we analyze three such models that are based on experimental results to determine how they affect populations of synapses converging onto a postsynaptic neuron. We focus on two basic features: stability and competition.

Stability is a property of the distribution of synaptic weights arising from an STDP model, and we will distinguish three cases: unstable, partially stable, and stable. In the unstable case, synaptic weights perpetually increase under STDP, unless some upper limit is imposed (in principle, weights could also decrease perpetually, but this is atypical). Although we briefly consider soft bounds to limit the range of synaptic weights, we primarily consider hard bounds. When hard limits are imposed on an unstable STDP model, the synaptic weights cluster tightly against the upper bound. Another more interesting case is partial stability, in which individual synaptic weights increase or decrease indefinitely, but the average of the weights across a synaptic population stays fixed. When hard bounds are imposed on a partially stable STDP model to limit the increases and decreases of individual synapses, the synaptic weights tend to cluster at either end of their allowed range, forming a U-shaped distribution [9]. Finally, when an STDP model is stable, no hard bounds need to be imposed, and synaptic weights form a unimodel distribution [10, 11, 12, 13]. We are interested in determining whether, and under what parameter values, different multi-spike STDP models lead to stable, partially stable or unstable synaptic weight distributions.

The impact of STDP on the weights of synapses onto a postsynaptic neuron depends on correlations between their presynaptic spike patterns. This can be studied by dividing the inputs to a neuron into two groups, one with correlated presynaptic activity and the other with uncorrelated presynaptic spiking. In this context, competition refers to the propensity of either a correlated or an uncorrelated group of synapses to gain control of the postsynaptic spiking, while the other group become less influential, both as a consequence of STDP. In cases that we call “Hebbian”, the synapses with correlated input become stronger than those with uncorrelated input. In other “anti-Hebbian” cases, the reverse occurs and the correlated synapses become weaker than the uncorrelated. We are interested in whether synaptic plasticity is Hebbian or anti-Hebbian, by this definition, in various multi-spike STDP models and for different parameter values of those models.

The three multi-spike STDP models that we consider were proposed on the basis of different experimental results. In the “suppression model”, inspired by experimental results in cortical slices, the plasticity-inducing effect of each pre- or postsynaptic spike is suppressed by preceding spikes [6, 14]. The “triplet model”, inspired by experiments in hippocampal slices, includes the effect of neighboring pre-post pairings as well as depression exerted by preceding presynaptic spikes and potentiation by preceding postsynaptic spikes [15]. The third model we consider, the “NMDAR-based model”, is phenomenologically based on the kinetics of the N-Methyl-D-Aspartate receptor [16]. This model was proposed before experimental results on multi-spike effects in STDP were available, and hence it was not explicitly aimed at accounting for multi-spike interactions, unlike the first two models. However, as we show in the Results section, it demonstrates a rich repertoire of multi-spike interactions, and can behave similar to either of the first two models depending on its parameters. Therefore, we feel it deserves to be considered as a multi-spike STDP model, even though it may not have been intended as such initially.

We begin by reviewing results for pair-based STDP to establish our approach and introduce ways of characterizing the effects of plasticity. We then apply this approach and these characterizations to the multi-spike STDP models. For each case, we first consider the parameters originally proposed for the model, and then systematically explore a range of parameter values to evaluate stability and competition. In light of the results obtained, we conclude by discussing relationships between the models at the biophysical level, and the computational implications of each model at the synaptic population level.

## Results

To study the effect of different STDP models on synaptic weights, we simulated a single spiking neuron receiving *N*_ex_ excitatory and *N*_in_ inhibitory presynaptic spike trains with Poisson statistics at rates *r*_pre_ and *r*_in_, respectively. The strengths of the excitatory synapses, denoted by *w*_*i*_ with *i* = 1,2,…,*N*_ex_, change according to STDP, while the strengths of inhibitory synapses remain constant. Since we focus on the distribution of excitatory synaptic strengths, we treat them as random variables collectively labeled with *w*. To examine the different forms of stability of each STDP model, we check whether the steady-state distribution of synaptic strengths is bounded without imposing external limits, or whether the increase or decrease of the weights is stopped only when they hit a boundary. We distinguish between the partially stable and unstable cases by computing the evolution of the average of the synaptic weights, which reaches a fixed point only in the partially stable case. Fully stable STDP is characterized by a fixed point for the average rate and bounded deviations for the strengths of individual synapses about this mean. As a probe of synaptic competition, we induce correlations to a subset of the excitatory inputs (Methods) and check whether STDP causes the synapses corresponding to correlated and uncorrelated subsets to compete for control of the postsynaptic firing. This also allows us to determine whether the effect of correlations is Hebbian or anti-Hebbian. In the following sections, we report the competitive behavior of the models in response to a fixed correlation coefficient of 0.2 induced in half of the synapses. In S1 Fig we also show results for a range of correlation coefficients. Except for few important exceptions that will be pointed out, the qualitative behavior of the models is not sensitive to the choice of correlation coefficient. Similarly, changing the proportion of correlated synapses to be more or less than half does not change the obtained results qualitatively. The neuronal and input parameters used in our simulations are given in Table 1.

**Table 1:** Neuronal, synaptic, and plasticity parameters.

Parameter	Symbol	Default value
Membrane time constant	*τ*_m_	20 ms
Spiking threshold	*V*_th_	*−*40 mv
Resting membrane potential	*V*_r_	*−*60 mv
Synaptic time constant	*τ*_s_	5 ms
Number of excitatory synapses	*N*_ex_	1000
Number of inhibitory synapses	*N*_in_	250
Inhibitory synaptic strength	*w*_in_	1 mv
Excitatory input rate	*r*_pre_	10 Hz
Inhibitory input rate	*r*_in_	10 Hz
Correlation coefficient *	c	0.2

* Correlation is only introduced for simulations in which synaptic competition is examined.

### Pair-based STDP

**Fig 1:**
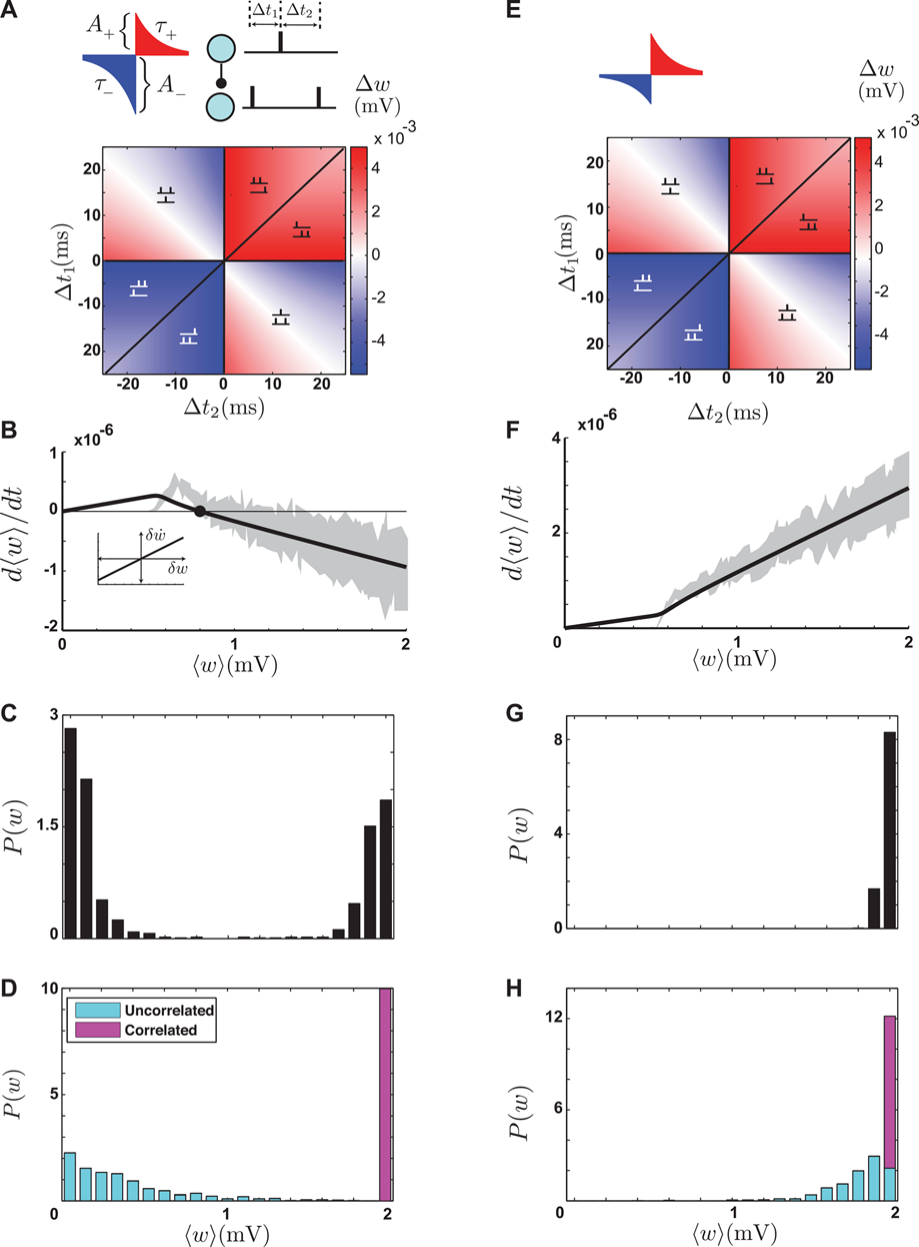
The pair-based STDP model. **A.** Top-left: The STDP window with *A*_+_ < *A*_−_. Top-right: a triplet of spikes composed of two pre-post pairs with intervals Δ*t*_1_ and Δ*t*_2_. Bottom: the amount of synaptic modification in response to triplets, which is symmetric in the pair-based model. **B.** The average drift induced by the pair-based model on a population of excitatory synapses converging onto a single postsynaptic neuron, when *A*_+_ < *A*_−_. The black curve is a numerical evaluation of Eq 2 and the gray area is the simulation results. The half-width of the gray area is the standard error. The filled circle is the stable fixed point. The inset shows the *w*-dependent drift (Eq 3) **C.** The steady state distribution of synaptic weights obtained by simulation when *A*_+_ < *A*_−_. **D.** The steady state distribution of weights when half of the synapses receive correlated input (magenta) and the other half receive uncorrelated input (cyan). When *A*_+_ < *A*_−_ correlated synapses are strengthened. **E-H.** The same as A-D, but for *A*_+_ > *A*_−_. Note that there is no stable fixed point in **F**, and that all the synapses are pushed to the upper bound in **G** and **H**. For these simulations, the constants of the STDP model were *τ*_+_ = *τ*_−_ = 20 *ms*, *A*_+_ = 0.005 *mV* and *A*_−_ = 1.0.1 *A*_+_ in A-D and *A*_−_ = 0.005 *mV* and *A*_+_ = 1.0.1 *A*_−_ in E-H.

To explain our method for analyzing synaptic stability and competition and also to provide a benchmark of comparison for the multi-spike STDP models, we first examine a pair-based STDP model. In this model, synapses are modified only on the basis of the intervals between pairs of pre- and postsynaptic spikes. When a synapse receives a larger ensemble of spikes, such as triplets or quadruplets, plasticity is induced by the pre-post pairs within the ensemble independent of the higher-order structure of the ensemble. As stated in the introduction, similarly spaced “pre-post-pre” and “post-pre-post” triplets induce the same amount of synaptic modification in this model (Fig 1A and 1E). The parameters of the pair-based model include the maximum amounts by which synapses can be potentiated or depressed, *A*_+_ and *A*_−_, and the time constants for the potentiation and depression windows, *τ*_+_ and *τ*_−_ (Eq 10). These parameters also appear in the multi-spike models.

To quantify the average modification of a synapse under STDP, we first calculate the probability of a pairing interval Δ*t* for spikes arriving at the synapse and then average synaptic modification (Eq 10) over that probability. We assume that the pre- and postsynaptic spike trains are both Poisson. The rate of the presynaptic spike train takes the constant value *r*_pre_. The baseline rate of postsynaptic firing is denoted by *r*_post_ (Eq 8). When a postsynaptic action potential is generated, presynaptic spikes are equally likely to arrive at any later time because the postsynaptic spike has no effect on presynaptic activity. However, when a presynaptic spike arrives at a particular synapse, it transiently increases the postsynaptic firing rate by an amount proportional to the strength of that synapse (Eq 9). As a result, a postsynaptic action potential is more likely to be induced shortly after the arrival of a presynaptic spike. Including both the baseline rate and this brief enhancement, the average synaptic modification or the “drift” for a synaptic strength *w* is (see Methods)

$$\frac{dw}{dt}=\left(A_{+}\tau_{+}-A_{-}\tau_{-}\right)\;r_{\text{pre}}\;{\bar{r}}_{\text{post}}+\frac{A_{+}\tau_{+}\tau_{\text{s}}\;r_{\text{pre}}\;w}{\left(\tau_{\text{s}}+\tau_{+}\right)\left(V_{\text{th}}-V_{\text{r}}\right)\;\tau_{\text{m}}}.$$ (1)

The first term in this equation relates the change in synaptic strength of a particular synapse, *w*, to the average strength of all the excitatory synapses, ⟨*w*⟩, through the dependence of the baseline firing rate ${\bar{r}}_{\text{post}}$ on this average. This term is the same for all synapses, so we call it the “baseline drift”. The second term depends on the synaptic strength of the particular synapse being considered, and it arises from the transient increase of postsynaptic firing rate following a presynaptic spike at this synapse. We call it the “*w*-dependent drift”. The rate of change of the average of all the excitatory synaptic weights is given by the sum of the baseline drift and the average of the *w*-dependent drift,

$$\frac{d\langle w\rangle }{dt}=\left(A_{+}\tau_{+}-A_{-}\tau_{-}\right)\;r_{\text{pre}}\;{\bar{r}}_{\text{post}}+\frac{A_{+}\tau_{+}\tau_{\text{s}}\;r_{\text{pre}}\;\langle w\rangle }{\left(\tau_{\text{s}}+\tau_{+}\right)\left(V_{\text{th}}-V_{\text{r}}\right)\;\tau_{\text{m}}}.$$ (2)

The average synaptic strength in the steady-state is the values of ⟨*w*⟩ that sets the right side of Eq (2) to zero (i.e. a fixed point). One such fixed point occurs when all the synapses are zero (⟨*w*⟩ = 0). This makes the postsynaptic neuron silent (${\bar{r}}_{\text{post}}=0$) and sets both the baseline and average *w*-dependent drifts to zero. This state is uninteresting and simply reflects the fact that no plasticity occurs when the postsynaptic neuron is silent. If the synaptic strengths are not zero, the average *w*-dependent drift is always positive because presynaptic spikes always enhance postsynaptic firing. As a result, a nontrivial fixed point for the average synaptic weight can occur only if the baseline drift is negative (*A*_−_ *τ*_−_ > *A*_+_ *τ*_+_) so that it can cancel the *w*-dependent drift (Fig 1B, closed circle). This fixed point is stable, because the positive *w*-dependent drift dominates if the average weight is smaller that the fixed-point value, and the negative baseline drift dominates if it is larger. Mathematically, stability requires the slope of the average drift to be negative at the fixed point (Fig 1B), which always holds for the nontrivial fixed point of the pair-based model. In summary, the steady-state average synaptic strength in pair-based STDP has a stable nontrivial mean if the depression window is larger than the potentiation window (*A*_−_ *τ*_−_ > *A*_+_ *τ*_+_). This fixed point is unique, so the mean of the steady-state distribution of synaptic weights converges to this value regardless of its initial value.

The stability of the mean is not a sufficient condition for the steady-state distribution of synaptic strengths to be fully stable, each synapse must also have a stable deviation from the mean. The strength of a particular synapse can be expressed as *w* = ⟨*w*⟩ + *δw*, where *δw* is the deviation of the synapse from the mean. If the deviation tends to grow over time, the synapses will drift away from the mean and the distribution will be partially stable and U-shaped (bimodal). If the deviation tends to decrease, the synapses will cluster around the mean and the distribution will be stable and unimodal. Assuming that the mean synaptic strength is at steady-state and that the deviation of an individual synapse (out of a few thousand) does not alter the mean significantly, the change of the deviation over time is governed solely by the *w*-dependent drift and can be derived from Eq (1) as

$$\frac{d\delta w}{dt}=\frac{A_{+}\tau_{+}\tau_{\text{s}}\;r_{\text{pre}}\;\delta w}{\left(\tau_{\text{s}}+\tau_{+}\right)\left(V_{\text{th}}-V_{\text{r}}\right)\;\tau_{\text{m}}}.$$ (3)

Because the coefficient of *δw* in this equation is positive (Fig 1B, inset), the deviations tends to grow, and the final distribution of synaptic strengths for pair-based STDP is partially stable and U-shaped even though the mean is stable (Fig 1C, ref. [9]).

To check the accuracy of Eqs (2) and (3), we computed the synaptic drift by averaging the amount of induced synaptic modification in simulations lasting 0^3^ s of simulated time, without implementing synaptic modification (Fig 1B, gray shade). In general, the agreement is good; the discrepancy between the analytic and simulation results at low average synaptic strengths is due to the fact that our approximation for the transient postsynaptic firing rate (Eq 9) is only accurate when the mean excitatory input is significantly larger than the mean inhibitory input. In the parameter regime where the potentiation window is larger than the depression window (Fig 1E–1H), the mean synaptic weight only has the trivial and unstable zero fixed point (Fig 1F), so the distribution is unstable and all of the synaptic strengths grow until they hit the upper bound, regardless of their initial values (Fig 1G).

When the mean synaptic strength is stable and the *w*-dependent drift is positive, it is possible for STDP to discriminate between two groups of synapses based on the degree of correlation in their presynaptic spike trains. If the spike trains arriving at one group of synapses are correlated and those of the other synapses are not, the correlated group induces a larger transient increase in the postsynaptic firing rate and hence a larger *w*-dependent drift. Therefore the correlated group is more likely to become stronger than the mean, and the uncorrelated group tends to become weaker to maintain the balance around the mean (Fig 1D). This results in a Hebbian competition among the synapses [9]. On the other hand, when there is no stable mean, all the synapses tend to grow regardless of their correlation and no competition takes place, although the correlated synapses still end up stronger than the uncorrelated group (Fig 1H). Therefore, the condition for Hebbian competition through pair-based STDP is the existence of a stable mean, i.e. *A*_−_ *τ*_−_ > *A*_+_ *τ*_+_, which is equivalent to partial stability. Importantly, as long as the steady-state mean of the synaptic strength is within the allowed range, this condition is not changed by modifying the lower or upper bounds of the synaptic strengths. This is also the case for the multi-spike models discussed in the following sections.

### The triplet model

**Fig 2:**
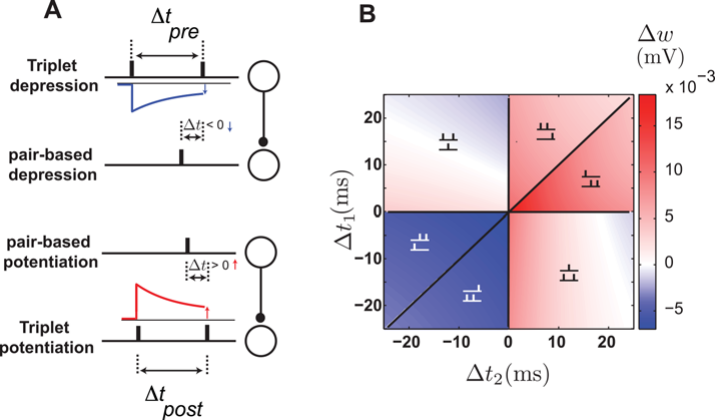
The triplet model. **A.** Schematic illustration of spike interactions in the triplet model in which previous presynaptic spikes induce extra depression (top) and previous postsynaptic spikes induce extra potentiation (bottom). **B.** Plasticity due to triplets of spikes: pre-post-pre triplets induce depression or weak potentiation (top left), and post-pre-post ordering induces mostly potentiation (bottom right). This figure is based on parameters fit to hippocampal data (Table 2).

Experimental results on synapses in hippocampal cultures reveal a marked asymmetry in the plasticity induced by post-pre-post and pre-post-pre spike sequences, in contrast to the predictions of the pair-based model (Fig 1A and 1E). Post-pre-post sequences induce potentiation, and pre-post-pre has little or no effect [7]. In addition, in experiments on cortical synapses, the balance between potentiation and depression shifts toward potentiation when the frequency of pre-post pairing events increases, another property not captured by pair-based STDP [17]. These results motivated Pfister & Gerstner [15] to propose the triplet model, which takes into account interactions of spikes beyond pre-post pairings. In addition to the effect of pre-post pairings, the triplet model includes additional depression due to previous presynaptic spikes and additional potentiation from earlier postsynaptic spikes (Fig 2A). This is accomplished through a presynaptic depression variable and a postsynaptic potentiation variable assigned to each synapse (Eq 11). In the absence of incoming presynaptic spikes, the presynaptic depression variable decays exponentially with time constant *τ*_pre_. Likewise, the value of postsynaptic potentiation variable decreases exponentially in the absence of postsynaptic spikes with time constant *τ*_post_. When a presynaptic spike reaches the synapse, the presynaptic depression variable abruptly increases by the amount *A*_pre_, and when a postsynaptic spike occurs, the postsynaptic variable increases by *A*_post_ (Eq 12). This is how the triplet model accounts for the asymmetry of synaptic modification in response to triplets. For a pre-post-pre triplet, the first presynaptic spike induces extra depression on the synapse, while for a post-pre-post triplet the first postsynaptic spike induces extra potentiation (Fig 2B). The triplet model that we consider sums the contributions of all previous pre- and postsynaptic spikes as well as all pre-post pairings (all-to-all). Pfister & Gerstner [15] also provided a version of the triplet model based only on nearest neighboring spikes, but the qualitative behavior of both versions is similar.

**Table 2:** Original parameters of the multi-spike STDP models used to generate Figs 2B, 4B, 7B and 9.

	Triplet	Suppression	NMDAR-based
*A*_+_	5.3 *×* 10^*−*3^ mV	1.3 *×* 10^*−*2^ mV	10^*−*3^ mV
*A*_−_	3.5 *×* 10^*−*3^ mV	5.1 *×* 10^*−*3^ mV	10^*−*3^ mV
*A*_pre_	0	–	–
*A*_post_	8 *×* 10^*−*3^ mV	–	–
$A_{f}^{\text{up}}$	–	–	1
$A_{f}^{\text{dn}}$	–	–	0.5
$A_{M}^{\text{up}}$	–	–	0.7
$A_{M}^{\text{dn}}$	–	–	0.7
*τ*_+_	16.8 ms	13.3 ms	–
*τ*_−_	33.7 ms	34.5 ms	–
*τ*_pre_	–	28 ms	–
*τ*_post_	40 ms	88 ms	–
$\tau_{f}^{\text{up}}$	–	–	300 ms
$\tau_{f}^{\text{dn}}$	–	–	300 ms
$\tau_{M}^{\text{up}}$	–	–	600 ms
$\tau_{M}^{\text{dn}}$	–	–	600 ms
*θ*^up^	–	–	0.7
*θ*^dn^	–	–	0.35

As we did for pair-based STDP, we can derive equations governing the evolution of the mean synaptic strengths and deviations around the mean for individual synaptic weights. The average values of the presynaptic depression and postsynaptic potentiation variables, obtained from substituting rates for spikes in Eq 12, are *A*_pre_ *τ*_pre_ *r*_pre_ and $A_{\text{post}\;}\tau_{\text{post}}\;{\bar{r}}_{\text{post}}$. Using these values and averaging the synaptic modification (Eq 11) over the probability of pre-post pairings, the drift of the mean of the synaptic weights in the triplet model is

$$\begin{array}{lll} \frac{d\langle w\rangle }{dt} & = & A_{+}\tau_{+}r_{\text{pre}}\;{\bar{r}}_{\text{post}}+A_{\text{post}}\;\tau_{\text{post}}\;\tau_{+}r_{\text{pre}}\;{\bar{r}}_{\text{post}}^{2}-A_{-}\;\tau_{-}r_{\text{pre}}\;{\bar{r}}_{\text{post}}-A_{\text{pre}}\;\tau_{\text{pre}}\;\tau_{-}r_{\text{pre}}^{2}\;{\bar{r}}_{\text{post}}\\ & & +\frac{\left(A_{+}+A_{\text{post}}\;\tau_{\text{post}}\;{\bar{r}}_{\text{post}}\right)\;\tau_{+}\tau_{\text{s}}\;r_{\text{pre}}\;\langle w\rangle }{\left(\tau_{\text{s}}+\tau_{+}\right)\left(V_{\text{th}}-V_{\text{r}}\right)\;\tau_{\text{m}}}. \end{array}$$ (4)

As in the pair-based model, the last term in this equation is the *w*-dependent drift and the other terms make up the baseline drift. The dynamics of deviations of individual synapses from the mean is governed by the *w*-dependent drift, so

$$\frac{d\delta w}{dt}=\frac{\left(A_{+}+A_{\text{post}}\;\tau_{\text{post}}\;{\bar{r}}_{\text{post}}\right)\;\tau_{+}\tau_{\text{s}}\;r_{\text{pre}}\;\delta w}{\left(\tau_{\text{s}}+\tau_{+}\right)\left(V_{\text{th}}-V_{\text{r}}\right)\;\tau_{\text{m}}}.$$ (5)

As in the pair-based model, the coefficient of *δw* is always positive, so individual weights will drift away from the mean for any choice of parameters, making individual synaptic weights unstable.

The parameters of the original model were fit by Pfister & Gerstner [15] separately to match experimental data from hippocampal cultures and cortical slices, resulting in two sets of parameters. Our simulation results indicate that, for both sets of parameters, the distribution of synaptic weights is unstable, so that all the synaptic weights cluster around the upper bound. In addition, no competition takes place between correlated and uncorrelated synapses with these parameter sets. This led us to consider properties of the triplet model for a range of parameter values. As in our discussion of pair-based STDP, we study the triplet model when pair-based potentiation is larger than pair-based depression (*A*_−_ = 0.005 mV, *A*_+_ = 1.01*A*_−_) and when pair-based depression is larger than pair-based potentiation (*A*_+_ = 0.005 mV, *A*_−_ = 1.01*A*_+_). In each case, we varied the ratio between postsynaptic potentiation and presynaptic depression (*A*_post_/*A*_pre_) systematically, while keeping *A*_pre_ constant at 0.001 mV.

**Fig 3:**
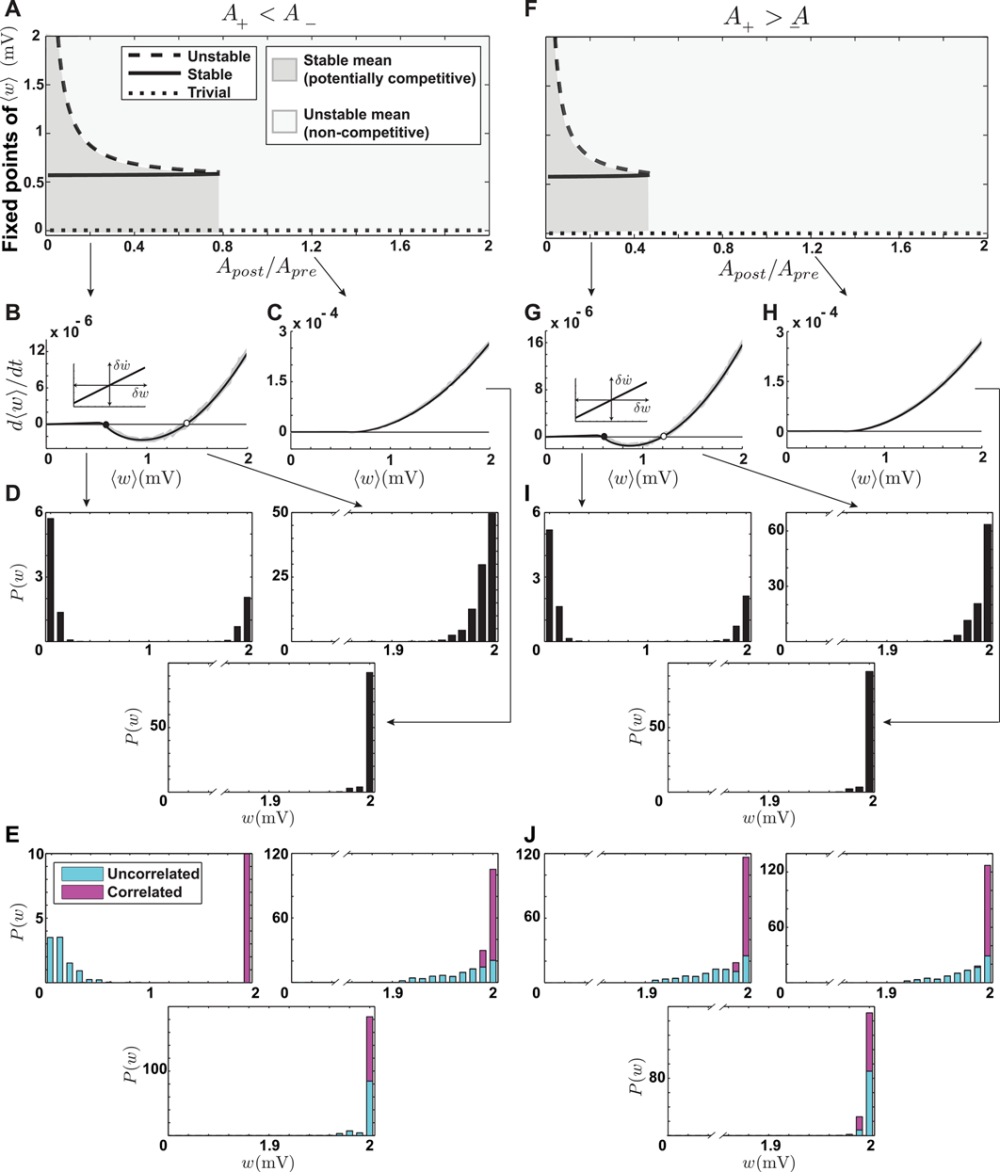
Stability and competition in the triplet model. **A-E.** Pair-based depression is larger than pair-based potentiation. **A.** Fixed points of ⟨*w*⟩ as functions of the ratio between postsynaptic potentiation and presynaptic depression parameters (*A*_post_/*A*_pre_). When *A*_post_/*A*_pre_ is small, two nontrivial fixed points exist, one stable and one unstable. At higher values, they collide and disappear. When a stable fixed point exists (solid curve), the model is potentially competitive (dark gray area). **B.** Average drift of the weights, when *A*_post_/*A*_pre_ = 0.2. The gray area shows simulation results, and the solid curve is obtained from Eq (4). The filled circle depicts the stable fixed point and the open circle the unstable fixed point. The inset shows the *w*-dependent drift near the stable fixed point. **C.** Average drift of the weights when *A*_post_/*A*_pre_ = 1.2. The average weight has no nontrivial fixed points. **D.** Distribution of synaptic weights obtained from simulation. With parameters as in B and an initial mean of 0.4 mV, the final distribution is U-shaped (left). With an initial mean of 1.6, the final distribution clusters around the upper bound (right). Using parameters as in C, the final distribution also clusters around the upper bound (bottom). **E.** Synaptic competition for the parameters and initial values used in corresponding panels of D. Hebbian competition occurs only when the mean weight is stable and its initial value is below the unstable fixed point (left). **F-J.** Same as A-E, but when pair-based potentiation is larger than pair-based depression. **F.** The nontrivial fixed points disappear at lower values of *A*_post_/*A*_pre_ than in A, making the potentially competitive region smaller than in A (dark gray area). **G-J.** The same as B-E, but with pair-based potentiation larger than pair-based depression. Because the stable and unstable fixed points are close (G), competition does not occur even in the presence of a stable fixed point for the mean weight (J, left). For this figure, the time constants of presynaptic depression and postsynaptic potentiation were *τ*_pre_ = *τ*_post_ = 40 ms, and the pair-based parameters of the model were the same as the pair-based model in Fig 1.

We first examine the fixed points of the mean synaptic weight (Fig 3A and 3F). When *A*_post_/*A*_pre_ is small, the average synaptic weight has two nontrivial fixed points (Fig 3B and 3G). The first is stable (Fig 3B and 3G, filled circle) and the second is unstable (Fig 3B and 3G, open circle). The appearance of the unstable fixed point in the triplet model is due to the dependence of the postsynaptic potentiation on the postsynaptic firing rate. This added potentiation increases when the mean synaptic weight increases, eventually overcoming the combined effect of presynaptic and pair-based depression. The existence of two fixed points makes the steady-state distribution of synaptic weights sensitive to the initial distribution. If the mean of the initial distribution is greater than the unstable fixed point, the distribution will be unstable and all of the weights will be pushed toward the upper bound (Fig 3D and 3I, right). If the mean of the initial distribution is lower than the unstable fixed point, the mean of the steady-state distribution converges to the stable fixed point and individual weights drift away from the mean toward the lower and upper bounds, resulting in partial stability and a U-shaped distribution similar to the pair-based model (Figs 3D and 2I, left). When *A*_post_/*A*_pre_ reaches a critical value, the two fixed points coalesce and annihilate each other, and only the trivial unstable fixed point remains (Fig 3C and 3H). In this case, regardless of the initial distribution, the final distribution is unstable and tightly clustered near the upper bound (Fig 3D and 3I, bottom).

As we argued in the case of the pair-based model, synaptic competition can only take place when the steady-state distribution has a nontrivial stable mean and is partially stable. In the triplet model, when *A*_post_/*A*_pre_ is relatively small and the initial mean synaptic weight is lower than the unstable fixed point, this condition is fulfilled (Fig 3A and 3F, dark gray areas; Fig 3E, left). However, if the stable and unstable fixed points are too close together, there is no guarantee of synaptic competition (Fig 3J, left). The reason for this is that when a subset of the synaptic inputs are correlated, presynaptic spikes tend to arrive in tandem and induce large transients in the postsynaptic firing rate, causing large fluctuations in the mean synaptic weight. This can cause the mean synaptic strength to fluctuate beyond the unstable fixed point, destabilizing the weight distribution. As a result, the parameter regime for synaptic competition in the triplet model is highly restricted to the region of small *A*_post_/*A*_pre_ with *A*_+_ < *A*_−_ (Fig 3A, dark gray). Even within this small region, if the correlation coefficient among the correlated synapses is high, competition does not take place and all the synapses tend to the upper bound (S1 Fig), due to the fluctuations mentioned above. Thus, it is not surprising that the original parameters obtained by Pfister & Gerstner [15] did not lead to competitive synaptic plasticity. In summary, the novel properties of the triplet model, as compared to the pair-based model, are the sensitivity to the initial distribution of weights and a tighter parameter range for Hebbian competition.

### The suppression model

**Fig 4:**
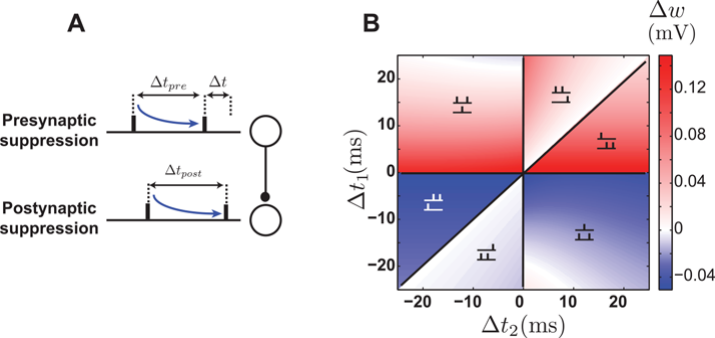
The suppression model. **A.** Schematic illustration of spike interactions in the suppression model, in which the effect of the presynaptic spike in a pair is suppressed by a previous presynaptic spike (top), and the effect of the postsynaptic spike is suppressed by a previous postsynaptic spike (bottom). **B.** Plasticity in the suppression model induced by triplets of spikes: pre-post-pre triplets induce potentiation (top left), and post-pre-post triplets induce depression (bottom right).

Plasticity experiments in cortical slices using triplets of spikes showed different effects than the hippocampal results. In the synapses of the visual cortex of rats, pre-post-pre triplets induce potentiation whereas post-pre-post triplets induce depression [6]. These results led Froemke et al. [6] to propose the suppression model, in which plasticity is induce by nearest neighbor pre- and postsynaptic spikes. The plasticity is computed from the standard pair-based STDP curve, but the effect of the presynaptic spike in each pair is suppressed by previous presynaptic spikes and, similarly, the plasticity induced by the postsynaptic spike in each pair is suppressed by previous postsynaptic spikes (Fig 4A). The suppression is maximal immediately after each pre- or postsynaptic spike, and it decreases exponentially as the interval between consecutive pre- or postsynaptic spike increases (Eq 13). The suppression accounts for the asymmetry of synaptic modification in response to triplets. In the case of a pre-post-pre triplet, the first pair (pre-post) induces potentiation, but the amount of depression induced by the second pair (post-pre) is suppressed by the first presynaptic spike. For a post-pre-post triplet, the first pair (post-pre) induces depression, but the potentiation induced by the second pair (pre-post) is suppressed by the first postsynaptic spike (Fig 4B).

The parameters of the model were originally set to match the synaptic modification seen in the experiments (ref. [6]; Table 2). Our numerical simulations with these parameters show that the steady-state distribution is unstable and tightly clustered around the upper bound. When correlations are induced in half of the synaptic inputs, no competition takes place and all the weights are potentiated indiscriminately. To observe a range of behaviors of this model, we set the suppression time constants equal to the values given by Froemke et al. [6], namely *τ*_pre_ = 28 ms, *τ*_post_ = 88 ms. We also set the maximum potentiation and depression values equal (*A*_+_ = *A*_−_ = 0.005 mV and fixed the depression time constant (*τ*_−_ = 20 ms). We then varied the potentiation time constant *τ*_+_ to observe different behaviors of the model. Transitions to different behaviors can also be seen when changing other parameters (for example the ratio *A*_+_/*A*_−_), but our simulations showed that changing the ratio between the potentiation and depression time constants (*τ*_+_/*τ*_−_) reveals these transitions most clearly.

**Fig 5:**
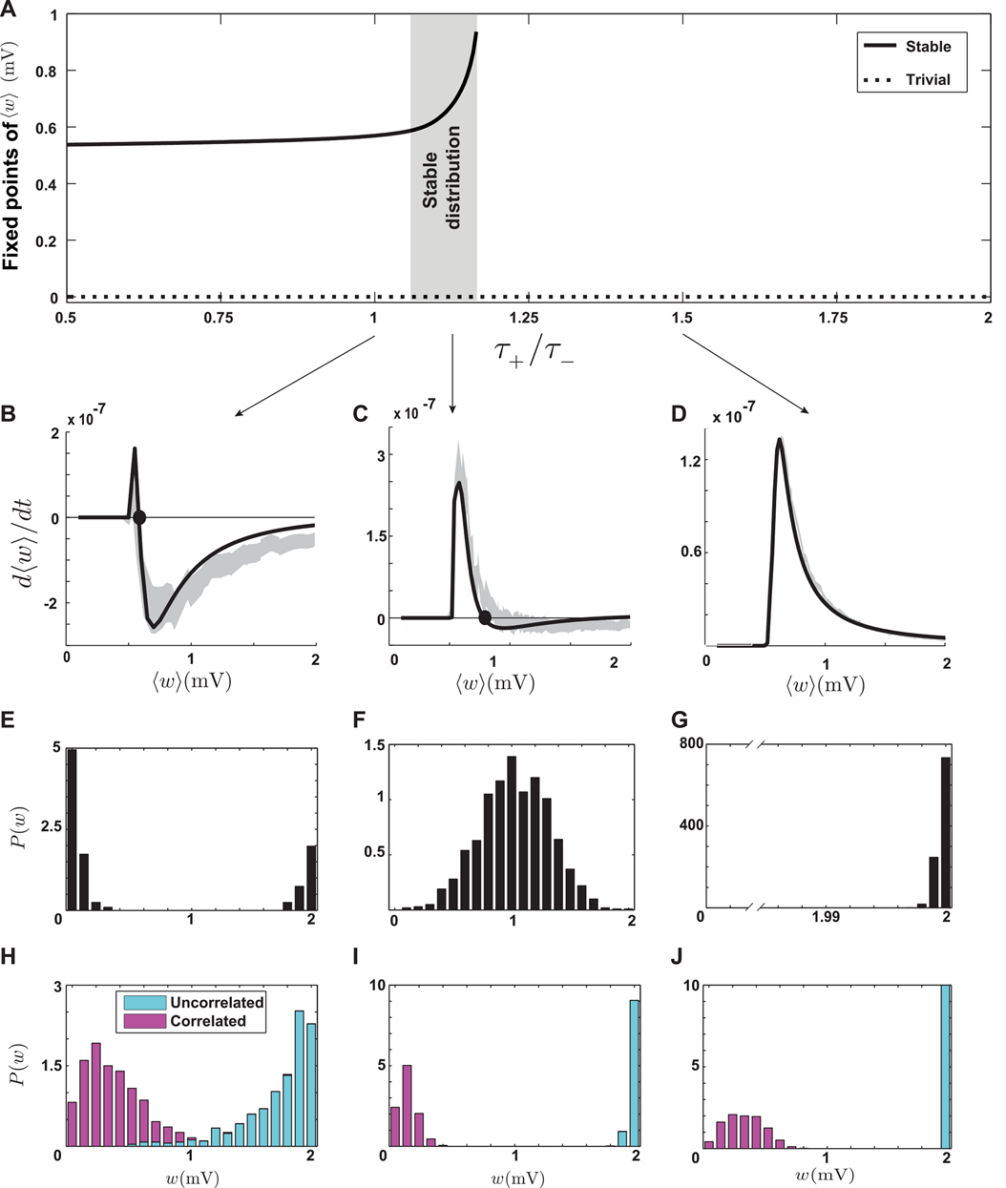
Stability and competition in the suppression model. **A.** Fixed points of ⟨*w*⟩ as functions of the ratio between the potentiation and depression time constants. The stable fixed point disappears beyond the critical value *τ*_+_/*τ*_−_ < 1.2. When the ratio approaches the critical value, the fixed point grows rapidly (gray area), leading to a stable distribution. **B.** The average drift when *τ*_+_/*τ*_−_ = 1. The solid curve shows the analytical result (Eq 6) and the boundaries of gray shading is obtained by simulations. The filled circle is the stable fixed point. **C.** The average drift when *τ*_+_/*τ*_−_ = 1.1. The stable fixed point moves to larger values than in B. **D.** The average drift when *τ*_+_/*τ*_−_ = 1.5. No nontrivial fixed point exists. **E.** The partially stable bimodal steady-state distribution of weights corresponding to the parameters of B. **F.** The stable steady-state distribution of weights corresponding to the parameters of C. **G.** The unstable steady-state distribution of weights clustered around the upper bound corresponding to the parameters of D, when no stable fixed point exists. **H-J.** Competition between correlated and uncorrelated synapses with parameter corresponding to E-G. The competition is anti-Hebbian in all cases.

Calculating the drift of synapses in the suppression model is more complicated than in the models considered above. We leave the details to S2 Appendix and report the results here. When *τ*_+_/*τ*_−_ < 1.2, the average synaptic weight has a stable nontrivial fixed point (Fig 5A–5C). For higher values of *τ*_+_/*τ*_−_, the nontrivial fixed point disappears and the average synaptic weigh has only the trivial zero fixed point (Fig 5A and 5D). For low *τ*_+_/*τ*_−_ values, the steady-state distribution of weights is partially stable and U-shaped, as in the case of the pair-based model (Fig 5E). However, for *τ*_+_/*τ*_−_ between 1.05 and 1.2, the value of the average synaptic weight grows rapidly (Fig 5A, gray area), and the steady-state distribution is stable and unimodal (Fig 5F), implying that the *w*-dependent drift is negative in this range. Because of the complexity of spike interaction in the suppression model, a complete characterization of the *w*-dependent drift is beyond our analytical calculations (S2 Appendix). However, features of the response of an integrate-and-fire neuron to a pair of presynaptic spikes in the context of the suppression model explain why the *w*-dependent drift becomes negative when the average synaptic weight is large.

**Fig 6:**
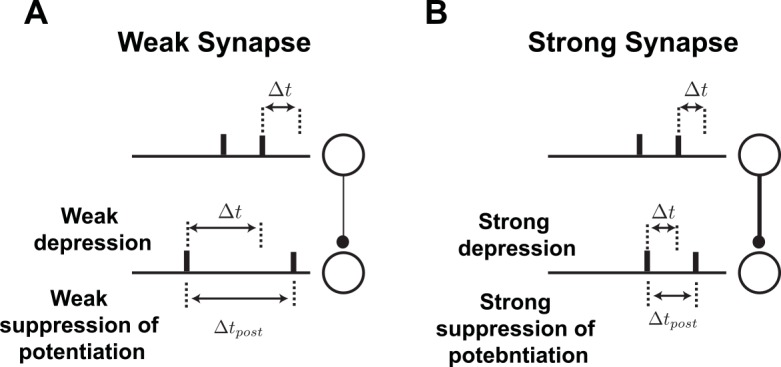
Response of a neuron to a pair of presynaptic spikes and its consequences in the suppression model. **A.** When the synapse is weak, the probability of a postsynaptic spike does not increase significantly from the baseline. The interval between postsynaptic spikes and also the pairing interval between the second presynaptic and the first postsynaptic spike are likely to be long. The result is a weak depression and also a weak suppression of potentiation. **B.** When the synapse is strong, the neuron is likely to fire in response to both presynaptic spikes, which results in strong depression and also strong suppression of potentiation.

Suppose that two presynaptic spikes arrive at a neuron in quick succession, and we want to analyze the role of the second spike in inducing plasticity under the suppression model (Fig 6). The second presynaptic spike participates in plasticity twice: once by pairing with the previous postsynaptic spike, and again by pairing with the next postsynaptic spike. When the strength of the synapse is low, the first presynaptic spike is not very likely to induce a postsynaptic action potential after its arrival, so the pairing interval between the second presynaptic spike and the preceding postsynaptic spike is typically long, which induce weak depression (Fig 6A). However, if the synapse is strong, the first presynaptic spike is likely to induce a postsynaptic action potential, and its pairing interval with the second presynaptic spike is then short, inducing strong depression (Fig 6B). In addition, because of the high probability of postsynaptic firing in response to both presynaptic spikes, the interval between the induced postsynaptic spikes is short, which strongly suppresses the potentiation caused by pairing the second presynaptic spike with its following postsynaptic spike. Therefore, depression dominates over potentiation in the suppression model when synapses are strong. When this happens, deviations to even higher values lead to depression. This explains why *w*-dependent drift is negative when the average synaptic weight is large, which occurs when *τ*_+_/*τ*_−_ approaches the critical value 1.2 (Fig 5A, gray area).

When half of the synapses receive correlated spike trains and the other half uncorrelated inputs, a distinctive features of the suppression model is that anti-Hebbian competition takes place: the uncorrelated synapses become strong and the correlated ones weak (Fig 5H–5J). This is the result of postsynaptic suppression. When correlated presynaptic spikes arrive, they tend to induce a postsynaptic spike shortly after their arrival. This makes the interval between the induced postsynaptic action potential and the previous spike shorter than for the postsynaptic response to uncorrelated input. As a result, potentiation is suppressed for correlated synapses, and they eventually lose the competition with uncorrelated ones. In analogy with what was described in the previous paragraph, correlated inputs are similar to inputs with strong synapses and, in either case, the high probability of postsynaptic spiking makes the *w*-dependent drift negative. In summary, the characteristic properties of suppression model are anti-Hebbian competition and stability of the synaptic distribution when the mean synaptic strength is large.

### The NMDAR-based model

The NMDAR-based model [16] was proposed as an explanation for the original STDP experiments of Markram et al. [4], and it predates both the triplet and suppression models and the data that inspired them. Nevertheless, as we will see below, it has features that resemble both of these models, and it is sensitive to spike interactions beyond pre-post pairings. The original version of the NMDAR-based model [16] includes the dynamics of the probability of presynaptic vesicle release. We focus on a simpler version that only models the modification of synaptic strengths by pre- and postsynaptic spikes [18].

**Fig 7:**
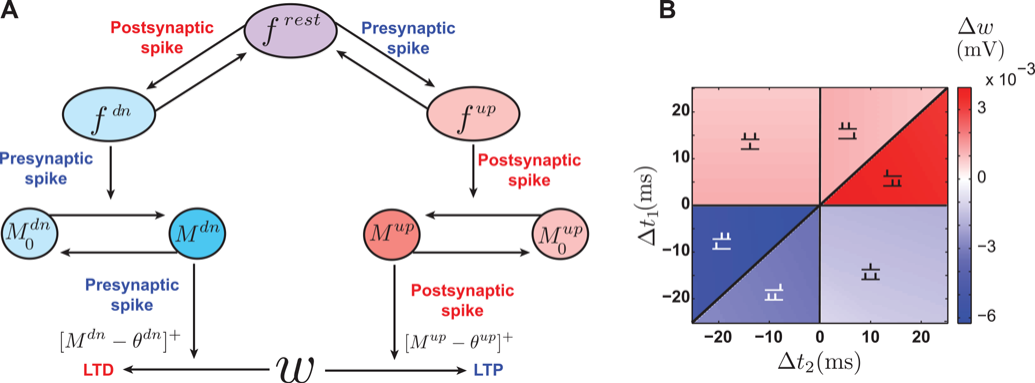
The NMDAR-based model. **A.** Schematic illustration of spike interactions in the NMDAR-based model. The presynaptic spike up-regulates *f*^rest^, activates *M*^dn^ and depresses the synapse. The postsynaptic spike down-regulates *f*^rest^, activates *M*^up^ and potentiates the synapse. **B.** Plasticity in the NMDAR-based model due to triplets of spikes with parameters as in Table 2. The effect is asymmetric, with pre-post-pre triplets inducing potentiation (top left) and post-pre-post depression (bottom right).

In the NMDAR-based model, the NMDAR is assumed to have three states, rest, up and down. Each incoming presynaptic spike moves a portion of the NMDARs in the rest state into the up state, and each postsynaptic spike transitions a portion of the rest-state NMDARs into the down state. The NMDAR decays back to the rest state exponentially in the absence of spikes (E 14). In accord with the molecular kinetics of NMDARs [19, 20], the rest state can be interpreted as an NMDAR that is not bound to glutamate and is blocked by Mg^2+^, the up state as an NMDAR that is bound to glutamate but blocked by Mg^2+^, and the down state as an NMDAR that is not bound to glutamate but has had its Mg^2+^ block removed by a postsynaptic spike. The model also has two second messengers, called “up” and “down” messengers, which mediate potentiation and depression, respectively. These can be in either active or inactive states. When a presynaptic spike arrives, a fraction of the inactive down messengers transition to the active state. Likewise, when a postsynaptic spike reaches the synapse, it moves a portion of the inactive up messengers into their active state. The messengers decay back to their inactive states in the absence of spikes (Eq 15). Finally, upon arrival of a presynaptic spike, the synapse is depressed proportional to the amount of active down messenger, provided that this is larger than a threshold *θ*^dn^. Similarly, each postsynaptic spike causes the synapse to potentiate proportional to the amount of active up messenger provided that it is larger than a threshold *θ*^up^ (Eq 16). Thus, the presynaptic spike plays three roles in this model: it moves resting NMDARs into the up state, it activates the down messenger, and it induces depression. The postsynaptic spike also has three roles: it transitions resting NMDARs into the down state, activates the up messenger, and induces potentiation (Fig 7A).

A key feature of the NMDAR based model is that preceding spikes decease the amount of available resting NMDARs available to upcoming spikes. This implements a mechanism akin to the suppression model, in which previous spikes suppress the effect of subsequent spikes. The roles of the second messengers are quite similar to those of the presynaptic depression and postsynaptic potentiation variables in the triplet model in that both integrate the effects of pre- and postsynaptic spiking to modify depression and potentiation. In fact, if we assume that the spikes have access to an unlimited pool of resting NMDARs and messengers, the NMDAR-based model is equivalent to the triplet model.

Given the multi-spike interactions in the NMDAR-based model, it is not surprising that it responds asymmetrically to triplets of spikes. Our numerical simulations using the parameters provided by Senn et al. [16] (Table 2) show that the synaptic modification in response to triplets in this model is qualitatively similar to that of the suppression model (Fig 7B). The simulations also show that, with the parameters provided by Senn et al. [16], the steady-state distribution is unstable and tightly clustered around the upper bound. When correlations are induced in half of the synaptic inputs, no competition takes place and all the weights are potentiated indiscriminately.

**Fig 8:**
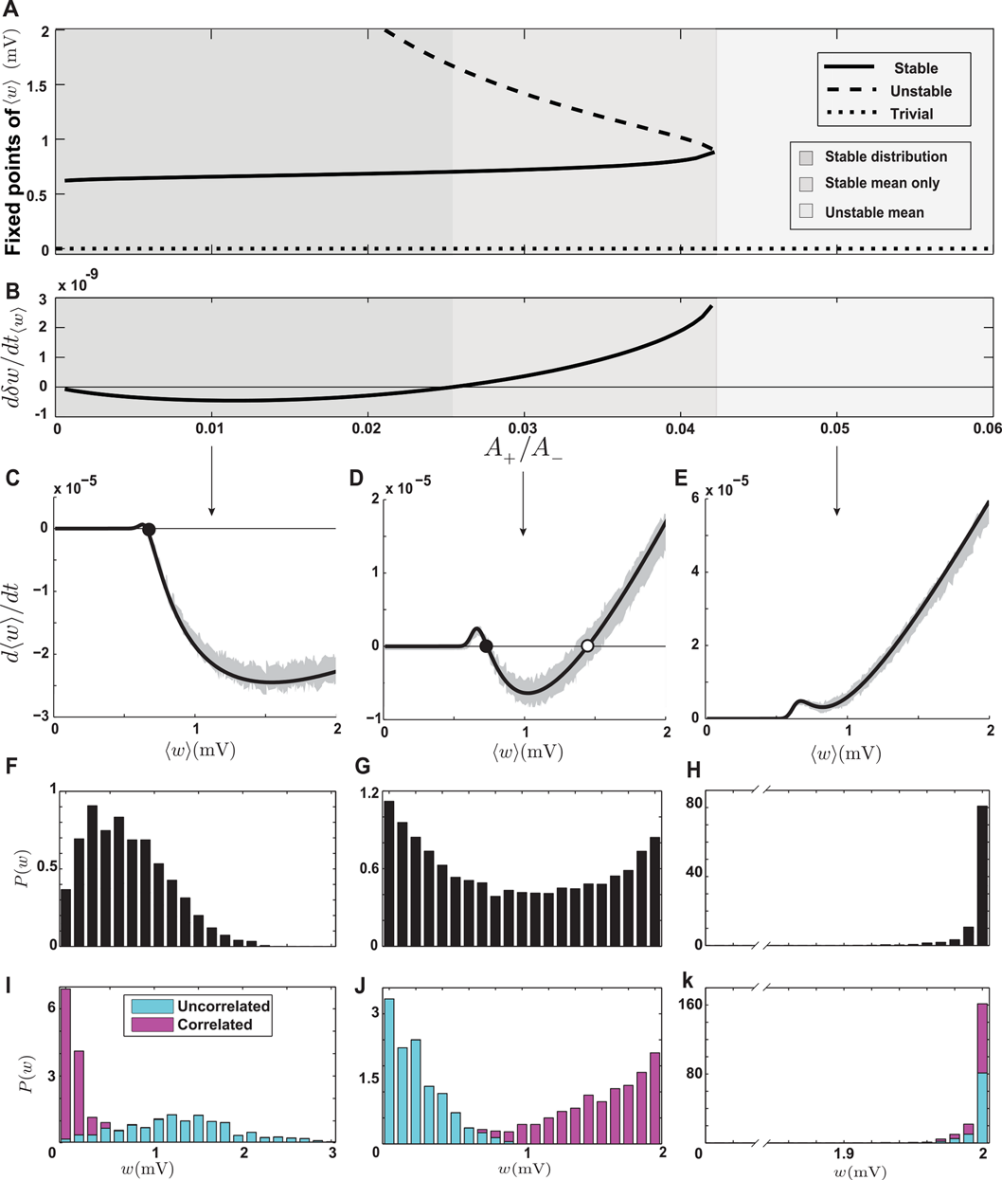
Stability and competition in the NMDAR-based model. **A.** Fixed points of ⟨*w*⟩ as functions of the ratio between the maximum potentiation and depression parameters. When *A*_+_/*A*_−_ is smaller than 0.042, two nontrivial fixed points exist. At higher values, they collide and disappear. **B.** The *w*-dependent drift at the stable fixed point, as a function of *A*_+_/*A*_−_, which changes sign at *A*_+_/*A*_−_ = 0.025. In the dark gray region, a stable fixed point exists and the *w*-dependent drift is negative. In the light gray region a stable fixed point exists and the *w*-dependent drift is positive, and in the white region there is no stable fixed point. **C-E.** The average drift when *A*_+_/*A*_−_ is 0.01, 0.03 and 0.05, respectively. Filled circles represent stable fixed points and the open circle an unstable fixed point. The gray shading is the result of simulations, and the solid curve is the analytical result. **F-H.** The steady-state distributions corresponding to the parameters in C-E. **I-K.** Synaptic competition between correlated and uncorrelated synapses corresponding to the parameters in C-E. Parameters are $A_{f}^{\text{up}}=A_{f}^{\text{dn}}=A_{M}^{\text{up}}=A_{M}^{\text{dn}}=0.1$, and the time constants are as in Table 2.

To examine the spectrum of behaviors in the NMDAR-based model, we calculated the synaptic drift (S3 Appendix). Interesting transitions into different regimes occur when the threshold of the up messenger is larger than that of the down messenger (*θ*^dn^ = 0.2, *θ*^up^ = 0), and the ratio between maximum potentiation and maximum depression (*A*_+_/*A*_−_) is varied (Fig 8). All other parameters of the model are held constant at equal values for potentiation and depression components, and the time constants are set to the values provided by Senn et al. [16] (Table 2). When *A*_+_/*A*_−_ is smaller than a critical value (0.042), the average synaptic weight has both stable and unstable nontrivial fixed points. At the critical value, these two fixed points coalesce and disappear, and beyond the critical value the average synaptic weight has only the trivial fixed point at zero (Fig 8A). The sign of *w*-dependent drift also changes as *A*_+_/*A*_−_ varies. When *A*_+_/*A*_−_ is smaller than 0.025, the *w*-dependent drift is negative, and for larger ratios it is positive (Fig 8B). Taken together, three different behaviors are observed in the NMDAR-based model: 1) When a stable mean synaptic weight exists and *w*-dependent drift is negative (0 < *A*_+_/*A*_−_ < 0.025, Fig 8A–8B, dark gray area), the steady-state distribution of synaptic weights is stable and unimodal (Fig 8C and 8F). 2) When a stable mean synaptic weight exists and *w*-dependent drift is positive (0 < *A*_+_/*A*_−_ < 0.042, Fig 8A and 8B, light gray area), the steady-state distribution of synaptic weights is partially stable and U-shaped (Fig 8D and 8G). 3) When the mean synaptic weight has no stable fixed point (*A*_+_/*A*_−_ > 0.042), the steady state distribution is unstable, and it clusters near the upper bound (Fig 8E and 8H).

Synaptic competition is different in these three regions of the parameter space. When half of the input spike trains are correlated, the competition in the first region is anti-Hebbian because the *w*-dependent drift is negative and correlated synapses receive more depression (Fig 8I). It is Hebbian in the second region because the *w*-dependent drift is positive (Fig 8J). As in the triplet model, the closeness of the stable and unstable fixed points in this region makes synaptic competition elusive, such that when the correlation coefficient between the synapses is high, all the synapses tend to the upper bound and no competition takes place (S1 Fig). There is no competition in the third region because the mean is not stable (Fig 8K). In short, the distinguishing features of the NMDAR-based model compared to the pair-based model are the possibility of a stable synaptic distribution and anti-Hebbian competition when the maximum depression is significantly larger than the maximum potentiation.

### STDP with soft bounds

In previous sections, we imposed so-called hard bounds on the synaptic strengths to confine them between zero and a maximum allowed value (*w*_max_). It is also possible to confine the synapse by implementing soft bounds, that is, by making the maximum depression and potentiation weight-dependent so that when a synaptic strength approaches the bounds, its rate of change gradually decreases. This can be done by multiplying *A*_+_ and *A*_−_ by 1 − *w*/*w*_max_ and *w*/*w*_max_ respectively. In the case of the triplet model, the presynaptic depression and postsynaptic potentiation variables should be also multiplied by 1 − *w*/*w*_max_ and *w*/*w*_max_ respectively, because they appear as potentiation and depression factors as well.

**Fig 9:**
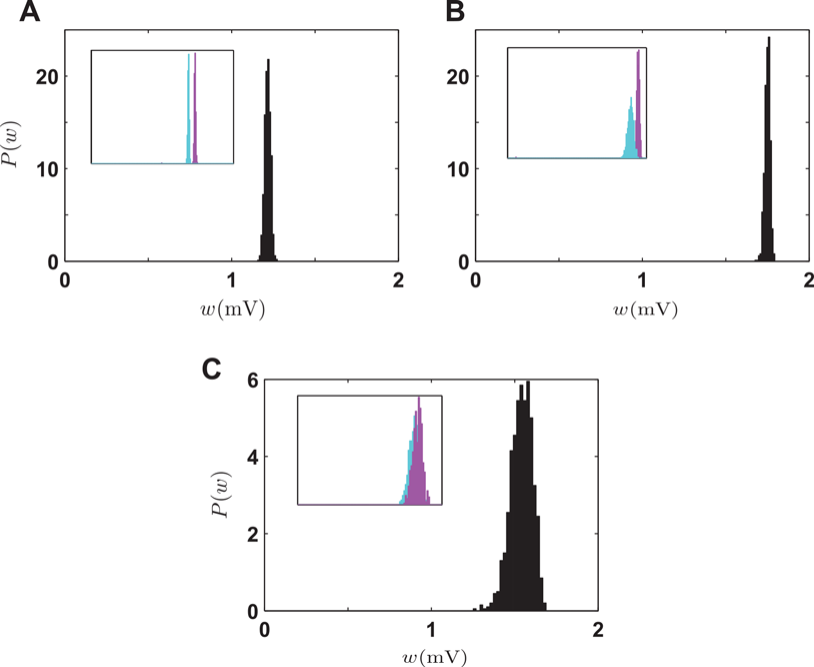
Stability and competition in multi-spike STDP models with soft bounds. **A.** Steady-state distribution of synaptic strengths in the triplet model with soft bounds. **B.** Steady-state distribution of synaptic strengths in the suppression model with soft bounds. **C.** Steady-state distribution of synaptic strengths in the NMDAR-based model with soft bounds. Insets: steady-state distribution of weights when half of the synapses receive correlated input (magenta) and the other half receive uncorrelated input (cyan). In each case, the original parameters (Table 2) are used.

The steady-state distribution of synaptic strengths is stable and unimodal for all three multi-spike STDP models with soft bounds (Fig 9). This behavior is robust and holds for a wide range of parameters (we only show simulation results for the original parameters (Table 2) in each model). The soft bounds weaken synaptic competition drastically, so than the distributions of correlated and uncorrelated synapses are close to each other (Fig 9, insets). As has been shown for pair-based STDP [10, 11], soft bounds turn STDP into a homeostatic plasticity mechanism with minimal sensitivity to the correlation structure of the external input.

## Discussion

The main focus of this study has been on synaptic stability and competition, two desirable but often conflicting features of activity-dependent plasticity rules [21]. Our analytical tool for assessing these properties was calculating the drift of a population of synapses under each multi-spike STDP model. This method has been applied to the pair-based STDP model in a number of previous studies. The pair-based model with hard bounds was shown to produce a partially stable U-shaped steady-state distribution of weights and Hebbian competition that favors correlated synapses over uncorrelated ones [9, 22, 23]. On the other hand, the pair-base model with soft bounds has been shown to have a stable steady-state distribution at the expense of losing synaptic competition and sensitivity to input correlations [10, 11]. Our analysis can be viewed as a reconfirmation of these results of the pair-based models and an extension into the domain of multi-spike STDP models.

Our goal has not been to identify a superior model among the different options. Rather, we have highlighted the largely overlooked consequences of implementing these models at the population level. Table 3 summarizes the results of our survey of stability and competition in multi-spike STDP models. Like the pair-based model, the triplet model produces a partially stable steady-state distribution of synaptic weights and Hebbian synaptic competition. However, competition is observed only for a limited range of its parameters. The suppression model shows predominantly anti-Hebbian competition and a stable steady-state distribution of synaptic weights when the average weight is high. The NMDAR-based model displays both stable and partially stable steady-state distributions depending on the parameters, with anti-Hebbian competition in the former case and Hebbian in the latter.

**Table 3:** Summary of stability/plasticity in STDP models.

	Stability	Partial stability	Hebbian competition	Anti-Hebbian competition
Pair-based	-	✓	✓	-
Triplet	-	✓	✓	-
Suppression	✓	✓	-	✓
NMDAR-based	✓*	✓	✓	✓

* Stability only coexists with anti-Hebbian competition is this model.

Our results indicate that the dichotomy between stability and Hebbian competition, which is well characterized for pair-based STDP models, persists in multi-spike STDP models. However, anti-Hebbian competition can coexist with full synaptic stability for at least some parameter regimes in the suppression and NMDAR-based models. Conflict exists between stability and Hebbian competition because, for such competition to take place, correlated synapses, which induce a large transient increase in postsynaptic firing rate, should be strengthened. This property undermines full stability of the synaptic distribution because it creates a positive feedback loop in which strong synapses, which also induce large transient increases in postsynaptic firing, become even stronger. Thus, it is not surprising that this conflict persists in more elaborate multi-spike models. Anti-Hebbian competition, on the other hand, involves weakening of correlated synapses that induce large transients in the postsynaptic activity, and so should be compatible with full stability of the synaptic distribution.

The dichotomy between synaptic competition and stability is a specific form of the general stability/plasticity dilemma [24]. Every form of plasticity faces the challenge of maintaining the balance between forming new memories through modification of synaptic strengths, and preserving old synaptic configurations to maintain old memories. A more specific aspect of this challenge is that Hebbian (or anti-Hebbian) competition among synapses in a network is a powerful mechanism for shaping and modifying neural activity based on the properties of the inputs to the network. However, unless changes in synaptic strength are stabilized appropriately, the level of activity in a neural circuit can grow or shrink in an uncontrolled way [25]. Therefore, it is highly desirable, from a computational point of view, to find a biologically plausible model that reconciles synaptic stability with competition. A number of solutions have been proposed for harmonizing stability and competition in pair-based STDP. One solution is interpolating between hard and soft bounds to obtain a middle ground that can harbor both synaptic competition and stability, which is obtained over a limited parameter range [12]. Another solution, based on a small temporal shift in the STDP window, can stabilize the distribution of synaptic weights while maintaining competitiveness [13]. This shift has a similar effect in the triplet model [13].

To search the parameter space of the models for different stability/plasticity interplays, we systematically varied the balance between potentiation and depression parameters in each multi-spike STDP model. However, for each model, a fixed set of parameters was originally proposed to match experimental results. Our parameter changes may cause the response profile of the model to deviate from its originally fitted form. This can be justified because both the temporal spread and the magnitude of potentiation and depression vary considerably as a function of the location of a synapse [14, 26, 27]. Therefore, each parameter set in our analyses and numerical simulations could coincide with the characteristics of the STDP window at a particular location on the dendritic tree.

Although all of the models we considered were proposed on the basis of experimental observations of synaptic modification, their effect on a population of synapses onto a postsynaptic neuron can be quite different. As mentioned above, one useful computational aspect of STDP is its ability to implement Hebbian learning and to functionally organize neural circuits. None of the three multi-spike models generated Hebbian competition when the original fitted parameters were used. Moreover, using these parameters, all three models produced an unstable distribution of weights tightly clustered near the upper bound of their allowed range. Given the observed broad distribution of synaptic weights in vitro [28, 29, 30] this is implausible. As it is possible to construct several phenomenological models that explain a given experimental data set, it seems reasonable to use the effects of plasticity at the population level (evaluated through simulations or analytical calculations) as a criterion for selecting a model. This criterion works particularly against model such as triplet STDP, because of its limited capacity for inducing competition among synapses at the population level even with altered parameter values, even though the model accounts for isolated experimental results satisfactorily.

**Fig 10:**
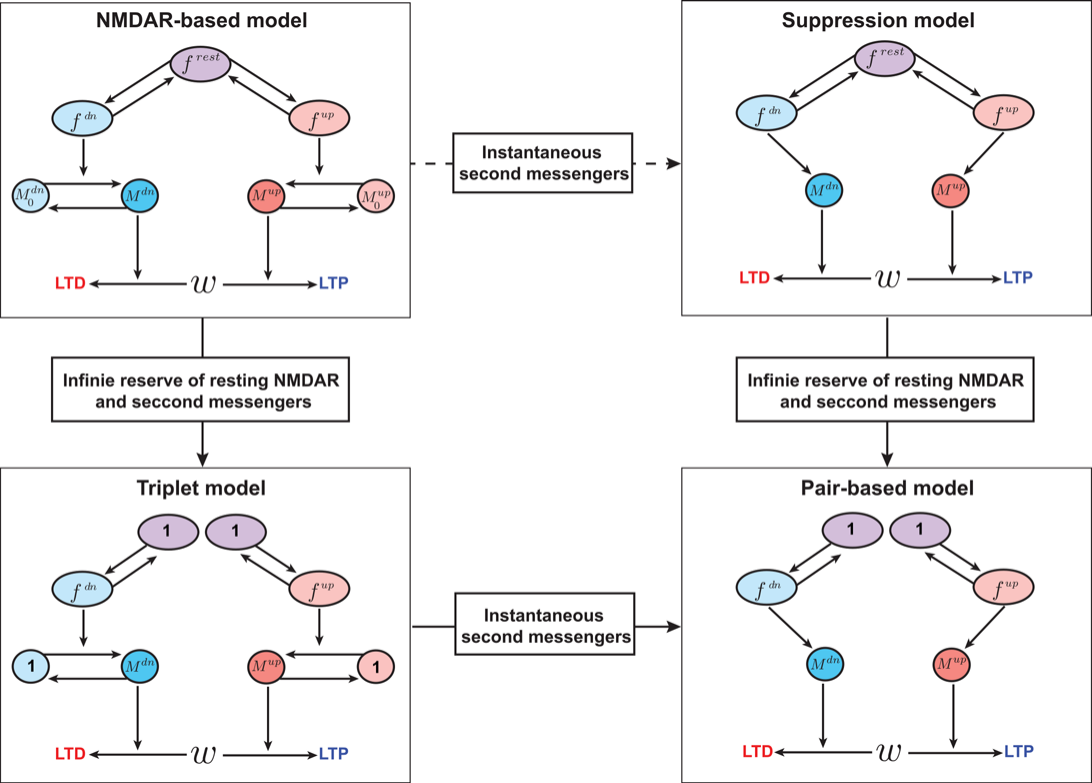
Relationships between the three multi-spike STDP models. If the second messengers activate instantaneously, the NMDAR-based model is qualitatively equivalent to the suppression model (top, right). If there exists an infinite reservoir of resting NMDARs and inactive second messengers, the NMDAR-base model reduces to the triplet model (bottom-left). If both assumptions are fulfilled, the NMDAR-based model reduces to the pair-based model (bottom- right).

Finally a natural question is whether the STDP models we have considered are interrelated in any way, or whether it is possible to unite them in a single framework. The triplet and suppression models were motivated by different experimental data sets that showed opposite synaptic modification in response to triplets (ref. [6] vs. ref. [7]). However, the NMDAR-based model, which is phenomenologically closer to the molecular machinery involved in synaptic modification, can match the effects of either of these models, depending on the parameters used. Moreover, from the biophysical viewpoint, the other two models can be considered limiting versions of the NMDAR-based model through different simplifying assumptions about its components (Fig 10). If the second messengers activate instantaneously, the NMDAR-based model is qualitatively equivalent to the suppression model. The consumption of the limited pool of resting NMDARs by conversion into up or down states implements the suppressive effect of the preceding pre- or postsynaptic spike on the upcoming pre-post interaction. On the other hand, if there exists an infinite reservoir of resting NMDARs and inactive second messengers, the NMDAR-base model reduces to the triplet model. If both assumptions are fulfilled, the NMDAR-based model reduces to the simple pair-based model. This leads to the possibility that both the triplet and suppression models may arise from a single biophysical mechanism that involves NMDARs [31], but under different conditions for the speed of conformational changes and abundance of second messengers.

## Models

### Neuronal and synaptic models

We used a leaky integrate-and-fire (LIF) model neuron in our numerical simulations. The membrane potential of the LIF neuron obeys

$$\tau_{\text{m}}\frac{dV}{dt}=\left(V_{\text{r}}-V\right)+I_{\text{ex}}-I_{\text{in}},$$ (6)

where *τ*_m_ is the membrane time constant, *V*_r_ is the resting potential, *I*_ex_ is the excitatory input and *I*_in_ the inhibitory input. Although these inputs appear as currents, they are actually measured in units of the membrane potential (mV) because a factor of the membrane resistance has been absorbed into their definition. When the membrane potential *V* reaches the firing threshold *V*_th_, the neuron fires an action potential and the membrane potential resets to the resting value *V*_r_. The numerical values of all parameters are given Table 1.

Each presynaptic action potential arriving at an excitatory or inhibitory synapse induces an instantaneous jump in the corresponding synaptic input (*I*_ex_ or *I*_in_), which decays exponentially between the input action potentials. The time course of the synaptic inputs can thus be expressed as

$$I_{\text{ex}}(t)=\sum_{i=1}^{N_{\text{ex}}}w_{i}\sum_{t_{i}^{k}\leq t}\exp(\frac{t_{i}^{k}-t}{\tau_{\text{s}}}),I_{\text{in}}(t)=w_{in}\sum_{i=1}^{N_{\text{in}}}\sum_{t_{i}^{k}\leq t}\exp\left(\frac{t_{i}^{k}-t}{\tau_{\text{s}}}\right),$$ (7)

where *w*_*i*_ is the weight for excitatory synapse *i*, *w*_*in*_ is the common fixed weight for all *N*_in_ inhibitory synapses, and $t_{i}^{k}$ is the time of the *k*-th action potential at synapse *i*. The sums over presynaptic spike times are limited to spikes that arrive prior to the time *t*. The synaptic time constant *τ*_s_ = 5 ms is taken to be the same for excitatory and inhibitory synapses. The excitatory synaptic strengths, labeled collectively as *w*, are modified by STDP.

If the rate of the excitatory and inhibitory inputs is *r*_pre_ and *r*_in_ respectively, the average firing rate of the LIF neuron can be approximated as [32]

$${\bar{r}}_{\text{post}}={\left(\tau_{\text{m}}\sqrt{\pi }\int_{\frac{V_{\text{r}}-\mu }{\sigma }+\alpha }^{\frac{V_{th}-\mu }{\sigma }+\alpha }\mathrm{dx exp}\left(x^{2}\right)(1+erf(x))\right)}^{-1},$$ (8)

where

$$\mu =\left(N_{\text{ex}}\;r_{\text{pre}}\;\langle w\rangle -N_{\text{in}}\;r_{\text{in}}\;w_{\text{in}}\right)\tau_{\text{s}}\;\text{and}\;\sigma^{2}=\frac{\left(N_{\text{ex}}\;r_{\text{pre}}\;{\left\langle w\right\rangle }^{2}+N_{\text{in}}\;r_{\text{in}}\;w_{\text{in}}^{2}\right)\tau_{\text{s}}^{2}}{\tau_{\text{m}}},$$

with ⟨*w*⟩ denoting the average value of the excitatory synaptic weights. The parameter $\alpha =\left|\zeta \left(1/2\right)\right|\sqrt{\tau_{\text{s}}/2\tau_{\text{m}}}$, where *ζ*, is a correction to account for the nonzero synaptic decay constant. The arrival of a presynaptic spike increases the firing rate of the postsynaptic neuron transiently. For an LIF neuron in the case where the average excitatory input dominates over the inhibitory input, the firing rate after the arrival of a presynaptic spike at time *t*_0_ can be approximated as ([33]; see S1 Appendix)

$$r_{\text{post}}\left(t\right)\approx {\bar{r}}_{\text{post}}+\frac{w\;\exp\left(-\frac{t-t_{0}}{\tau_{\text{s}}}\right)}{\left(V_{\text{th}}-V_{\text{r}}\right)\tau_{\text{m}}}\;\Theta \left(t-t_{0}\right),$$ (9)

where *w* is the strength of the synapse through which the presynaptic spike arrived, and Θ is the Heaviside step function.

### Correlated spike trains

To study synaptic competition, we introduce correlations into half of the excitatory input spike trains. To generate Poisson spike trains with homogeneous pairwise (zero-lag) correlations, a “generating” spike train with rate *r*/*c* was first produced. The correlated spike trains were then obtained by trimming the generating spike train, that is, by randomly deleting spikes with probability 1 − *c*. The resulting spike trains all have rate *r*, and each pair is correlated with correlation coefficient *c* [34].

### The pair-based model

In pair-based STDP, a change of synaptic strength, Δ*w*, is induced by a pair of pre- and postsynaptic action potentials with time difference (pairing interval) Δ*t* = *t*_post_ − *t*_pre_. The functional relation between the synaptic modification and the pairing interval is

$$\Delta w=F\left(\Delta t\right)=\{\begin{array}{cc} A_{+}\;\text{exp}\left(-\Delta t/\tau_{+}\right) & \text{if}\;\Delta t\geq 0\\ -A_{-}\;\text{exp}\left(\Delta t/\tau_{-}\right) & \text{if}\;\Delta t<0. \end{array}$$ (10)

The positive parameters *A*_+_ and *A*_−_ specify the maximum potentiation and depression, respectively. We express the synaptic strengths in units of the membrane potential (mV), so *A*_+_ and *A*_−_ have mV units. The time constants *τ*_+_ and *τ*_−_ determine the temporal spread of the STDP window for potentiation and depression (Fig 1A and 1E). In our analysis, we assume that the spike pairings are all-to-all, meaning that all possible pre-post pairs, not only the nearest neighbor pairs, contribute to plasticity. However, the results we derive apply qualitatively to a pair-based model with a nearest-neighbor restriction as well.

### The triplet model

In the triplet model, synapses are modified on the basis of pre-post pairing events in a manner similar to the pair-based model (Eq 10) but, in addition, when a synapse is potentiated by a pre-post pairing (Δ*t* > 0), the postsynaptic potentiation variable *M*_post_ is added to the amount of the pair-based potentiation *A*_+_. Similarly, when a synapse is depressed by a paring event (Δ*t* < 0), the presynaptic depression variable *M*_pre_ is added to the pair-based depression *A*_−_. Thus,

$$\Delta w=F_{\text{trip}}\left(\Delta t\right)=\{\begin{array}{ll} \left[A_{+}+M_{\text{post}}\left(t-\epsilon \right)\right]\;\text{exp}\left(-\Delta t/\tau_{+}\right) & \text{if}\;\Delta t\geq 0\\ -\left[A_{-}+M_{\text{pre}}\left(t-\epsilon \right)\right]\;\text{exp}\left(\Delta t/\tau_{-}\right) & \text{if}\;\Delta t<0. \end{array}$$ (11)

The small parameter *ϵ* ensures that the values of *M*_pre_ and *M*_post_ just before their update by the pre- or postsynaptic spikes are used. The postsynaptic potentiation and presynaptic depression variables are governed by the equations

$$\frac{dM_{\text{pre}}}{dt}=-\frac{M_{\text{pre}}}{\tau_{\text{pre}}}+A_{\text{pre}}\underset{i}{\sum }\delta \left(t-t_{\text{pre}}^{\left(i\right)}\right)$$

$$\frac{dM_{\text{post}}}{dt}=-\frac{M_{\text{post}}}{\tau_{\text{post}}}+A_{\text{post}}\sum_{i}\delta \left(t-t_{\text{post}}^{\left(i\right)}\right),$$ (12)

where *δ*(*t*) is the Dirac delta function, and $t_{\text{pre}}^{\left(i\right)}$ and $t_{\text{post}}^{\left(i\right)}$ are the times of arrival of pre- and postsynaptic spikes respectively. This introduces four parameters into the model beyond those of the pair-based model: the time constants *τ*_pre_ and *τ*_post_ and the increments *A*_pre_ and *A*_post_.

### The suppression model

In the suppression model with time constants *τ*_pre_ and *τ*_post_, the change in a synaptic weight is determined by

$$\begin{array}{c} \Delta w=F_{\text{supp}}\left(\Delta t\right)=\left[1-\text{exp}\left(-\Delta t_{\text{pre}}/\tau_{\text{pre}}\right)\right]\;\left[1-\text{exp}\left(-\Delta t_{\text{post}}/\tau_{\text{post}}\right)\right]\\ \;\times \{\begin{array}{cc} A_{+}\;\text{exp}\left(-\Delta t/\tau_{+}\right) & \text{if}\;\Delta t\geq 0\\ -A_{-}\;\text{exp}\left(\Delta t/\tau_{-}\right) & \text{if}\;\Delta t<0, \end{array} \end{array}$$ (13)

where Δ*t*_pre_ is the interval between the presynaptic spike in the pair and its preceding presynaptic spike, and Δ*t*_post_ is the interval between the postsynaptic spike and its preceding spike. The suppression model introduces two new parameters beyond those of the pair-based model: the time constants *τ*_pre_ and *τ*_post_.

### The NMDAR-based model

The NMDAR-based model [16, 18] is based on the assumption that NMDARs can be in one of three different states: “rest”, “up” and “down”. The variables *f*^rest^, *f*^up^ and *f*^dn^ denote the fraction of NMDARs in each state respectively (*f*^rest^ + *f*^up^ + *f*^dn^ = 1). In the absence of pre- and postsynaptic spikes, the receptors in up and down states return to the rest state with time constants $\tau_{f}^{\text{up}}$ and $\tau_{f}^{\text{dn}}$ respectively. Each presynaptic spike up-regulates the receptors immediately after its arrival by an amount proportional to a parameter $A_{f}^{\text{up}}$, and each postsynaptic spike down-regulates the receptors proportional to a parameter $A_{f}^{\text{dn}}$. The dynamics of the NMDARs in the “up” and “down” states can be expressed as:

$$\frac{df^{\text{up}}}{dt}=-\frac{f^{\text{up}}}{\tau_{f}^{\text{up}}}+A_{f}^{\text{up}}f^{\text{rest}}\underset{i}{\sum }\delta \left(t-t_{\text{pre}}^{\left(i\right)}\right)$$

$$\frac{df^{\text{dn}}}{dt}=-\frac{f^{\text{dn}}}{\tau_{f}^{\text{dn}}}+A_{f}^{\text{dn}}f^{\text{rest}}\sum_{i}\delta \left(t-t_{\text{post}}^{\left(i\right)}\right),$$ (14)

where the sums run over all pre- ($t_{\text{pre}}^{\left(i\right)}$) or postsynaptic ($t_{\text{post}}^{\left(i\right)}$) spike times, indexed by *i*. In this and subsequent equations, we assume the convention that a quantity multiplying a *δ* function is evaluated immediately before the time when the argument of the *δ* function is zero.

The fraction of active second messenger *M*^up^ is increased by postsynaptic spikes proportional to the amount of up-regulated NMDARs *f*^up^ and the available inactive messengers 1 − *M*^up^. Likewise, the fraction of active second messenger *M*^dn^ is increased by presynaptic spikes proportional to the amount of down-regulated NMDARs *f*^dn^ and available inactive messenger 1 − *M*^dn^. In the absence of spikes, these second messenger fractions decay with time constants $\tau_{M}^{\text{up}}$ and $\tau_{M}^{\text{dn}}$, respectively. Thus,

$$\frac{dM^{\text{up}}}{dt}=-\frac{M^{\text{up}}}{\tau_{M}^{\text{up}}}+A_{M}^{\text{up}}\;f^{\text{up}}\;\left(1-M^{\text{up}}\right)\underset{i}{\sum }\delta \left(t-t_{\text{post}}^{\left(i\right)}\right)$$

$$\frac{dM^{\text{dn}}}{dt}=-\frac{M^{\text{dn}}}{\tau_{M}^{\text{dn}}}+A_{M}^{\text{dn}}\;f^{\text{dn}}\;\left(1-M^{\text{dn}}\right)\sum_{i}\delta \left(t-t_{\text{pre}}^{\left(i\right)}\right),$$ (15)

where the sums run over all pre- ($t_{\text{pre}}^{\left(i\right)}$) or postsynaptic ($t_{\text{post}}^{\left(i\right)}$) spike times. The parameters $A_{M}^{\text{up}}$ and $A_{M}^{\text{dn}}$ governing the magnitude of the changes in the messengers on spiking events. Finally, synaptic potentiation occurs in response to postsynaptic spikes and depends on the amount of *M*^up^, and synaptic depression occurs in response to presynaptic spikes depending on the amount of *M*^dn^, so that

$$\frac{dw}{dt}=A_{+}\;[M^{\text{up}}-\theta^{\text{up}}{]}^{+}\sum_{i}\delta \left(t-t_{\text{post}}^{\left(i\right)}-\epsilon \right)-A_{-}[M^{\text{dn}}-\theta^{\text{dn}}{]}^{+}\sum_{i}\delta \left(t-t_{\text{pre}}^{\left(i\right)}-\epsilon \right),$$ (16)

where *θ*^up^ and *θ*^dn^ are thresholds above which the corresponding messengers take part in plasticity, and [*x*]^+^ denotes the piece-wise linear threshold function [*x*]^+^ = *x* for *x* > 0 and zero otherwise. The small parameter *ϵ* is included because, in this case, we evaluate the factors multiplying the *δ* functions *after* the time of a spike, as required by the model.

## Supporting Information

S1 Appendix Calculating the causal increase in postsynaptic firing. (PDF)

S2 Appendix Calculating average weight modification for the suppression model. (PDF)

S3 Appendix Calculating average weight modification for the NMDAR-based model 4. (PDF)

S1 Fig Synaptic competition with different levels of correlation. (PDF)
