# Supplementary material for: Stability and Competition in Multi-spike Models of Spike-Timing Dependent Plasticity
Source: PLoS Comput Biol. 2016 Mar 3;12(3):e1004750. doi: 10.1371/journal.pcbi.1004750 (PMC4777380; doi:10.1371/journal.pcbi.1004750)
Supplement: S1 Appendix — (PDF) [file pcbi.1004750.s001.pdf]

S1 Appendix: Calculating the causal increase in postsynaptic firing

We first derive an approximate expression for the causal effect of presynaptic spikes on postsynaptic firing rate of the LIF neuron. Assuming that the excitatory input to the neuron is significantly larger than inhibitory input ($I_{\text{ex}} >> I_{\text{in}}$), the first term (leak) on the right-hand-side of equation (6) can be disregarded. The fluctuations of the input are also negligible in this regime ,and the neuron fires almost regularly with inter-spike-intervals $1/\bar{r}_{\text{post}}$. If we suppose that the neuron has fired the last postsynaptic spike at time $t = 0$ and, in addition to the baseline input, it receives a presynaptic spike at time t_{pre} via a synapse with strength w, the membrane potential at later times $t > t_{\text{pre}}$ can be approximated as:

$$V(t) \approx V_{\text{r}} + \frac{(I_{\text{ex}} - I_{\text{in}})\, t}{\tau_{\text{m}}} + w\, \frac{\tau_{\text{s}}}{\tau_{\text{m}}} \left[1 - \exp\left(\frac{t_{\text{pre}} - t}{\tau_{\text{s}}}\right)\right].$$

From the above equation, the relationship between the time of the incoming presynaptic spike t_{pre} and the time that the neuron fires its next postsynaptic spike after the presynaptic spike $\Delta t = t_{\text{post}} - t_{\text{pre}}$ can be expressed as

$$t_{\text{pre}} = f(\Delta t) = \frac{\tau_{\text{m}}\,(V_{th} - V_{\text{r}})}{I_{\text{ex}} - I_{\text{in}}} - w\, \frac{\tau_{\text{s}}}{I_{\text{ex}} - I_{\text{in}}} \left[1 - \exp\left(-\frac{\Delta t}{\tau_{\text{s}}}\right)\right] - \Delta t\,. \qquad \text{(S1)}$$

The probability of a presynaptic spike occurring somewhere within the typical inter-spike-interval of the postsynaptic neuron is the reciprocal of the duration of the interval, which is the baseline postsynaptic rate, so $P(t_{\text{pre}}) = \bar{r}_{\text{post}}$. The instantaneous firing rate of the neuron after the presynaptic spike is the probability density of Δt, which can be expressed as

$$\begin{aligned} r_{\text{post}}(\Delta t) &= P(t_{\text{pre}})\,|f'(\Delta t)| && \text{(S2)} \\ &= \bar{r}_{\text{post}} \left[1 + w\, \frac{\exp(-\Delta t/\tau_{\text{s}})}{I_{\text{ex}} - I_{\text{in}}}\right] \\ &\approx \bar{r}_{\text{post}} + w\, \frac{\exp(-\Delta t/\tau_{\text{s}})}{(V_{th} - V_{\text{r}})\tau_{\text{m}}}\,. && \text{(S3)} \end{aligned}$$

This is the equation (9) of the Methods section.
