# Supplementary material for: Stability and Competition in Multi-spike Models of Spike-Timing Dependent Plasticity
Source: PLoS Comput Biol. 2016 Mar 3;12(3):e1004750. doi: 10.1371/journal.pcbi.1004750 (PMC4777380; doi:10.1371/journal.pcbi.1004750)
Supplement: S2 Appendix — (PDF) [file pcbi.1004750.s002.pdf]

S2 Appendix: Calculating average weight modification for the suppression model

Equation (9) of Methods section enables us to calculate the probability density of the intervals between pre- and post synaptic spikes ($\Delta t = t_{\text{post}} - t_{\text{pre}}$). Assuming that the presynaptic spike train is Poisson, the probability density of Δt is

$$
\begin{aligned}
P(\Delta t) &= r_{\text{pre}}\, r_{\text{post}}(\Delta t) \exp(-\Delta t\, r_{\text{pre}}) \\
&= \begin{cases} r_{\text{pre}} \left[\bar{r}_{\text{post}} + w \dfrac{\exp(-\Delta t/\tau_{\text{s}})}{(V_{th} - V_{\text{r}})\tau_{\text{m}}} \right] \exp(-\Delta t\, r_{\text{pre}}) & \text{if } \Delta t \geq 0 \\ \\ r_{\text{pre}}\, \bar{r}_{\text{post}} \exp(-\Delta t\, r_{\text{pre}}) & \text{if } \Delta t < 0\,. \end{cases}
\end{aligned}
\tag{S4}
$$

When the neuron fires almost regularly, the interval between two consecutive postsynaptic spikes can be considered to be $1/\bar{r}_{\text{post}}$. However, if a presynaptic spike arrives before a postsynaptic spike ($\Delta t > 0$), it shortens the interval between the upcoming postsynaptic spike and the preceding one. In this case, the interval between consecutive postsynaptic spikes (Δt_{post}) can expressed as a function of Δt from equation (S1). Taken together,

$$
\Delta t_{\text{post}} = \begin{cases} \dfrac{1}{\bar{r}_{\text{post}}} \left[1 - w \dfrac{\tau_{\text{s}} \left(1 - \exp(-\Delta t/\tau_{\text{s}})\right)}{\tau_{\text{m}} \left(V_{th} - V_{\text{r}}\right)} \right] & \Delta t \geq 0 \\ \\ \dfrac{1}{\bar{r}_{\text{post}}} & \Delta t < 0\,. \end{cases}
\tag{S5}
$$

We now have all the components required to calculate the average weight modification under the suppression model (see equation 13). In this model, each nearest neighboring pre-post pair of spikes with pairing interval Δt induces potentiation or depression depending on the ordering of the pair. In addition, the previous pre- and postsynaptic spikes participate in plasticity depending on their temporal distance from the spikes in the pair (Δt_{pre} and Δt_{post} respectively) . Of the three intervals participating in suppression model, Δt_{pre} and Δt are stochastic variables, while Δt_{post} is simply a function of Δt. Therefore, we should average the weight modification over all possibles values of Δt and Δt_{pre}. Given the assumption that the postsynaptic spike fires almost regularly, $|\Delta t|$ cannot be longer than the length of a

typical postsynaptic ISI ($1/\bar{r}_{\text{post}}$). Also, in the case where the postsynaptic spike precedes the presynaptic one ($\Delta t < 0$), there is a lower limit on Δt_{pre}: it cannot be shorter than the pre-post interval $|\Delta t|$. By these considerations, the average weight change can be calculated as

$$\frac{d\langle w\rangle}{dt} = \int_{-1/\bar{r}_{\text{post}}}^{+1/\bar{r}_{\text{post}}} d\Delta t\, P(\Delta t) \int_{\max(0,-\Delta t)}^{\infty} d\Delta t_{\text{pre}}\, P(\Delta t_{\text{pre}})\, F_{supp}(\Delta t, \Delta t_{\text{pre}}, \Delta t_{\text{post}})$$

where F_{supp} is the weight modification (equation 13). Because the presynaptic spike train is assumed to be Poisson, $P(\Delta t_{\text{pre}})$ is the waiting time of the Poisson process, namely $r_{\text{pre}} \exp(-\Delta t_{\text{pre}}\, r_{\text{pre}})$. Substituting equations (S4) and (S5) in the above equation and keeping only terms up to first order in $\langle w\rangle$ results in

$$\begin{aligned}\frac{d\langle w\rangle}{dt} &= \mathcal{E}(\tau_{\text{post}})\, \bar{r}_{\text{post}}\, r_{\text{pre}} \left[\frac{A_+\, \tilde{\tau}_+\, \mathcal{E}(\tilde{\tau}_+)}{1 + r_{\text{pre}}\, \tau_{\text{pre}}} - A_-\, \tilde{\tau}_- \mathcal{E}(\tilde{\tau}_-) + A_-\, \hat{\tau}_- \mathcal{E}(\hat{\tau}_-)\right] \\ &\quad + \langle w\rangle \frac{A_+\, r_{\text{pre}}}{\tau_{\text{m}}\, \tau_{\text{post}}\, (V_{th} - V_{\text{r}})} \left[\frac{\hat{\tau}_+\, \mathcal{E}(\hat{\tau}_+)\Big((\tau_{\text{post}} - \tau_s)\mathcal{E}(\tau_{\text{post}}) + \tau_{\text{s}}\Big)}{1 + r_{\text{pre}}\, \tau_{\text{pre}}} \right. \\ &\quad \left. - \tau_{\text{s}}\, \tilde{\tau}_+\, \mathcal{E}(\tilde{\tau}_+)\Big(1 - \mathcal{E}(\tau_{\text{post}})\Big)\right] \qquad \text{(S6)}\end{aligned}$$

with $\mathcal{E}(\tau) = 1 - \exp(-1/(r_{\text{post}}\, \tau))$ and time constants defined as

$$\tilde{\tau}_+ = \frac{\tau_+}{1 + r_{\text{pre}}\, \tau_+} \qquad \hat{\tau}_+ = \frac{\tau_{\text{s}}\, \tau_+}{\tau_{\text{s}} + \tau_+ + \tau_{\text{s}}\, \tau_+}$$

$$\tilde{\tau}_- = \frac{\tau_-}{1 + r_{\text{pre}}\, \tau_-} \qquad \hat{\tau}_- = \frac{\tau_{\text{pre}}\, \tau_-}{\tau_{\text{pre}} + \tau_- + \tau_{\text{pre}}\, \tau_-}\,.$$

Equation (S6) is numerically evaluated in figure 5.
