# Supplementary material for: Stability and Competition in Multi-spike Models of Spike-Timing Dependent Plasticity
Source: PLoS Comput Biol. 2016 Mar 3;12(3):e1004750. doi: 10.1371/journal.pcbi.1004750 (PMC4777380; doi:10.1371/journal.pcbi.1004750)
Supplement: S3 Appendix — (PDF) [file pcbi.1004750.s003.pdf]

S3 Appendix: Calculating average weight modification for the NMDAR-based model

To calculate the average synaptic change in the NMDAR-base model, we first assume that the pre- and postsynaptic spike trains are independent, i.e. we disregard the causal effect of presynaptic spikes on postsynaptic firing. Afterwards, we include the causal relation. Assuming that the pre- and postsynaptic spike trains are Poisson, we can replace the δ functions in equations (14) by their corresponding rates (r_{pre} and $\bar{r}_{\text{post}}$) and, setting the right-hand-side equal to zero to obtain the mean values of the NMDAR state variables in up and down states, we find

$$\langle f^{\text{up}} \rangle = \frac{A_f^{\text{up}}\, \tau_f^{\text{up}}\, r_{\text{pre}}}{1 + A_f^{\text{up}}\, \tau_f^{\text{up}}\, r_{\text{pre}} + A_f^{\text{dn}}\, \tau_f^{\text{dn}}\, \bar{r}_{\text{post}}}$$

$$\langle f^{\text{dn}} \rangle = \frac{A_f^{\text{dn}}\, \tau_f^{\text{dn}}\, \bar{r}_{\text{post}}}{1 + A_f^{\text{up}}\, \tau_f^{\text{up}}\, r_{\text{pre}} + A_f^{\text{dn}}\, \tau_f^{\text{dn}}\, \bar{r}_{\text{post}}}\,. \tag{S7}$$

The mean values of the second messengers $\langle M^{\text{up}} \rangle$ and $\langle M^{\text{dn}} \rangle$ could be calculated using the same technique. However we use a different method for the second messengers that is equivalent to the above technique for the mean and also enables us to calculate the variance around the mean. At any instant t that we observe the variable $M^{\text{up}}(t)$, the last incoming postsynaptic spike has arrived Δt previously. The value of $M^{\text{up}}(t - \Delta t)$ has increased instantly in response to the postsynaptic spike and decayed afterwards so, according to equation (15),

$$M^{\text{up}}(t) = \Big(M^{\text{up}}(t-\Delta t) + A_M^{\text{up}}\, f^{\text{up}}(t-\Delta t)\,\big(1 - M^{\text{up}}(t-\Delta t)\big)\Big)\, \exp(-\Delta t/\tau_M^{\text{up}})\,. \tag{S8}$$

Assuming that the system is in equilibrium, the mean value of M^{up} should be the same at the times $t - \Delta$ and t. Moreover, because the postsynaptic spike train is assumed to be Poisson, the probability of an incoming postsynaptic spike Δt before time t is $\bar{r}_{\text{post}}\, \exp(-\Delta t\, \bar{r}_{\text{post}})$. By setting the values of M^{up} equal to the mean and averaging over all possible Δt values

we have

$$\langle M^{\rm up}\rangle = \Big(\langle M^{\rm up}\rangle + A_M^{\rm up}\,\langle f^{\rm up}\rangle\,\big(1-\langle M^{\rm up}\rangle\big)\Big)\int_0^\infty dt\,\bar r_{\rm post}\,\exp(-t\,\bar r_{\rm post})\exp(-t/\tau_M^{\rm up})\,,$$

which results in

$$\langle M^{\rm up}\rangle = \frac{A_M^{\rm up}\,\tau_M^{\rm up}\,\langle f^{\rm up}\rangle\,\bar r_{\rm post}}{1+A_M^{\rm up}\,\tau_M^{\rm up}\,\langle f^{\rm up}\rangle\,\bar r_{\rm post}}\,. \tag{S9}$$

As mentioned before, the same result could be obtained by replacing the δ functions in equation (15) with their corresponding rates and solving for the steady-state. However, the same argument as for equation (S8) can be used to calculate the variance of $M^{\rm up}$ around its mean. The square of $M^{\rm up}(t)$ can be obtained by squaring both sides of equation (S8). Assuming the mean square of $M^{\rm up}$ to be the same at t and $t-\Delta t$ and averaging over all possible Δt values, we find

$$\begin{aligned}\langle {M^{\rm up}}^2\rangle \;=\;& \Big[(1-A_M^{\rm up}\,\langle f^{\rm up}\rangle)^2\langle {M^{\rm up}}^2\rangle + 2\,(1-A_M^{\rm up}\,\langle f^{\rm up}\rangle)A_M^{\rm up}\,\langle f^{\rm up}\rangle\,\langle M^{\rm up}\rangle + (A_M^{\rm up})^2\,\langle f^{\rm up}\rangle^2\Big]\\ &\times\int_0^\infty dt\,\bar r_{\rm post}\,\exp(-t\,\bar r_{\rm post})\exp(-2t/\tau_M^{\rm up})\,,\end{aligned}$$

which can be used to calculate the variance of $M^{\rm up}$ as

$$\sigma_{\rm up}^2 = \langle {M^{\rm up}}^2\rangle - \langle M^{\rm up}\rangle^2 \approx \frac{\langle M^{\rm up}\rangle^2}{2\,\bar r_{\rm post}\,\tau_M^{\rm up}(1+A_M^{\rm up}\,\tau_M^{\rm up}\,\langle f^{\rm up}\rangle\,\bar r_{\rm post})}\,. \tag{S10}$$

Similarly, the mean and the variance of $M^{\rm dn}$ are

$$\begin{aligned}\langle M^{\rm dn}\rangle &= \frac{A_M^{\rm dn}\,\tau_M^{\rm dn}\,\langle f^{\rm dn}\rangle\,r_{\rm pre}}{1+A_M^{\rm dn}\,\tau_M^{\rm dn}\,\langle f^{\rm dn}\rangle\,r_{\rm pre}}\\ \sigma_{\rm dn}^2 &\approx \frac{\langle M^{\rm dn}\rangle^2}{2\,r_{\rm pre}\,\tau_M^{\rm dn}(1+A_M^{\rm dn}\,\tau_M^{\rm dn}\,\langle f^{\rm dn}\rangle\,r_{\rm pre})}\,. \end{aligned}\tag{S11}$$

We have ignored the variance of $f^{\rm up}$ and $f^{\rm dn}$ in the above calculations because their contribution in $\sigma_{\rm up}^2$ and $\sigma_{\rm dn}^2$ is insignificant.

The second messengers $M^{\rm up}$ and $M^{\rm dn}$ participate in potentiation and depression immediately after their abrupt increase due to the most recent pre- or postsynaptic spikes. Based on

equation (14), the sizes of these abrupt changes are $A_M^{\text{up}}\,\langle f^{\text{up}}\rangle(1-\langle M^{\text{up}}\rangle)$ and $A_M^{\text{dn}}\,\langle f^{\text{dn}}\rangle(1-\langle M^{\text{dn}}\rangle)$ respectively. Therefore, the mean value of the second messengers participating in potentiation and depression are

$$\mu^{\text{up}} = \langle M^{\text{up}}\rangle + A_M^{\text{up}}\,\langle f^{\text{up}}\rangle(1-\langle M^{\text{up}}\rangle)$$

$$\mu^{\text{dn}} = \langle M^{\text{dn}}\rangle + A_M^{\text{dn}}\,\langle f^{\text{dn}}\rangle(1-\langle M^{\text{dn}}\rangle) \tag{S12}$$

Finally, assuming the steady-state values of M^{up} and M^{dn} to be Gaussian variables with the above mean and variance, the average synaptic change according to equation (16) can be calculated as

$$\begin{aligned}\frac{d\langle w\rangle_0}{dt} &= \frac{A_+\,\bar{r}_{\text{post}}}{\sqrt{2\pi}\,\sigma_{\text{up}}}\int_{-\infty}^{+\infty} dx\,[x-\theta^{\text{up}}]^+\,\exp\left(-\frac{(x-\mu^{\text{up}})^2}{2\,\sigma_{\text{up}}^2}\right) \qquad \text{(S13)}\\ &\quad -\frac{A_-\,r_{\text{pre}}}{\sqrt{2\pi}\,\sigma_{\text{dn}}}\int_{-\infty}^{+\infty} dx\,[x-\theta^{\text{dn}}]^+\,\exp\left(-\frac{(x-\mu^{\text{dn}})^2}{2\,\sigma_{\text{dn}}^2}\right)\\ &= A_+\,\bar{r}_{\text{post}}\left(\frac{\sigma_{\text{up}}}{\sqrt{2\pi}}\,\exp\left(-\frac{(\mu^{\text{up}}-\theta^{\text{up}})^2}{2\,\sigma_{\text{up}}^2}\right)+\frac{\mu^{\text{up}}-\theta^{\text{up}}}{2}\,(1+\text{erf}\,(\frac{\mu^{\text{up}}-\theta^{\text{up}}}{\sqrt{2}\,\sigma_{\text{up}}}))\right)\\ &\quad -A_-\,r_{\text{pre}}\left(\frac{\sigma_{\text{dn}}}{\sqrt{2\pi}}\,\exp\left(-\frac{(\mu^{\text{dn}}-\theta^{\text{dn}})^2}{2\,\sigma_{\text{dn}}^2}\right)+\frac{\mu^{\text{dn}}-\theta^{\text{dn}}}{2}\,(1+\text{erf}\,(\frac{\mu^{\text{dn}}-\theta^{\text{dn}}}{\sqrt{2}\,\sigma_{\text{dn}}}))\right)\,.\end{aligned}$$

The subscript 0 on the right-hand-side denotes that we have not include the causal effect of presynaptic spikes on the postsynaptic spikes yet.

When a presynaptic spikes arrives, it transiently increases the postsynaptic firing rate proportional to the strength (w) of the synapse through which it arrived (equation 9, see also Appendix A). Because the amount of NMDARs in down state (f^{dn}) increases through postsynaptic spikes, we should take into account the effect of this transient postsynaptic rate increase on f^{dn}. Apart from transiently increasing the postsynaptic firing rate, the presynaptic spike has another effect on the NMDARs: upon is arrival, it consumes part of the pool of NMDARs at rest step (f^{rest}) and turns them into f^{up}, leaving less f^{rest} available for the upcoming postsynaptic spikes. Because the NMDARs decay back to the rest state over time, the shorter the interval between pre- and postsynaptic spikes, the less time f^{rest} has the

postsynaptic spike at its disposal to convert into f^{dn}. Considering this effect and averaging over the full range of intervals between the presynaptic spike and its induced postsynaptic spikes in equation (14), we find

$$\begin{aligned}\frac{d\langle f^{\text{up}}\rangle}{dt} &= -\frac{\langle f^{\text{up}}\rangle}{\tau_f^{\text{up}}} + A_f^{\text{up}}\,\langle f^{\text{rest}}\rangle\, r_{\text{pre}} \qquad \text{(S14)}\\ \frac{d\langle f^{\text{dn}}\rangle}{dt} &= -\frac{\langle f^{\text{dn}}\rangle}{\tau_f^{\text{dn}}} + A_f^{\text{dn}}\,\langle f^{\text{rest}}\rangle\, \bar{r}_{\text{post}} + w\frac{r_{\text{pre}}\,\tau_{\text{s}}}{\tau_{\text{m}}\,(V_{th}-V_{\text{r}})}\,A_f^{\text{dn}}\,\langle f^{\text{rest}}\rangle\,\frac{\tau_{\text{s}}+(1-A_f^{\text{up}})\,\tau_f^{\text{up}}}{\tau_{\text{s}}+\tau_f^{\text{up}}}\,.\end{aligned}$$

As expected, if we disregard the w-dependent term (i.e. ignoring the causal effect of the presynaptic spikes on postsynaptic firing) and solve for the steady-state, we retrieve the result of equation (S7). By keeping the w-dependent term in the above equations, the following w-dependent terms will be added to the steady-states calculated in equation (S7),

$$\begin{aligned}\langle f^{\text{up}}\rangle_w &= -w\,\frac{\tau_{\text{s}}\,A_f^{\text{dn}}\,\tau_f^{\text{dn}}\,\langle f^{\text{up}}\rangle^2\left(\tau_{\text{s}}+(1-A_f^{\text{up}})\,\tau_f^{\text{up}}\right)}{\tau_{\text{m}}\,A_f^{\text{up}}\,\tau_f^{\text{up}}\,(V_{th}-V_{\text{r}})(\tau_{\text{s}}+\tau_f^{\text{up}})}\\ \langle f^{\text{dn}}\rangle_w &= w\,\frac{\tau_{\text{s}}\,A_f^{\text{dn}}\,\tau_f^{\text{dn}}\,r_{\text{pre}}\,(1-\langle f^{\text{dn}}\rangle)^2\left(\tau_{\text{s}}+(1-A_f^{\text{up}})\,\tau_f^{\text{up}}\right)}{\tau_{\text{m}}\,(1+A_f^{\text{up}}\,\tau_f^{\text{up}}\,r_{\text{pre}})\,(V_{th}-V_{\text{r}})(\tau_{\text{s}}+\tau_f^{\text{up}})} \qquad \text{(S15)}\end{aligned}$$

The subscripts w denote the w-dependent contributions to the steady-state NMDAR ratios. Note that the contribution is negative for f^{up} and positive for f^{dn}. Intuitively, this implies that as the synapse gets stronger, the neuron fires more in response to the presynaptic spikes, hence a greater portion of NMDARs move to the down state and the share in the up state becomes smaller.

The second messenger M^{up} is also activated by postsynaptic spikes, therefore we should take into account the causal effect of presynaptic spikes on the postsynaptic rate in this case as well. Each presynaptic spike also increases the amount of f^{up} available for postsynaptic spikes according to equation (15). By taking these effects into account, including the w-dependence from equation (S15) and averaging over all possible pre-post intervals, the

following w-dependent terms will be added to the steady-state of $M^{\rm up}$ and $M^{\rm dn}$,

$$\begin{aligned}\langle M^{\rm up}\rangle_w &= w\,\frac{A_M^{\rm up}\,\tau_M^{\rm up}\,\tau_{\rm s}\,r_{\rm pre}}{\tau_{\rm m}\,(V_{th}-V_{\rm r})\,(1+A_M^{\rm up}\,\tau_M^{\rm up}\,\langle f^{\rm up}\rangle\,\bar{r}_{\rm post})^2}\\ &\quad\times\left(\langle f^{\rm up}\rangle+\langle f^{\rm rest}\rangle\,\frac{A_f^{\rm up}\,\tau_f^{\rm up}}{\tau_{\rm s}+\tau_f^{\rm up}}\right)\end{aligned}$$

$$\langle M^{\rm dn}\rangle_w = \frac{\langle f^{\rm dn}\rangle_w\;A_M^{\rm dn}\,\tau_M^{\rm dn}\,r_{\rm pre}}{(1+A_M^{\rm dn}\,\tau_M^{\rm dn}\,\langle f^{\rm dn}\rangle\,r_{\rm pre})^2}\,. \tag{S16}$$

The mean values in equation (S12) inherit the w-dependence from equations (S15) and (S16) in the form

$$\mu_w^{\rm up} = \left(1-A_M^{\rm up}\,\langle f^{\rm up}\rangle\right)\langle M^{\rm up}\rangle_w + A_M^{\rm up}\left(1-\langle M^{\rm up}\rangle\right)\langle f^{\rm up}\rangle_w$$

$$\mu_w^{\rm dn} = \left(1-A_M^{\rm dn}\,\langle f^{\rm dn}\rangle\right)\langle M^{\rm dn}\rangle_w + A_M^{\rm dn}\left(1-\langle M^{\rm dn}\rangle\right)\langle f^{\rm dn}\rangle_w\,. \tag{S17}$$

Finally, by inserting the above results into equation (S13) and keeping only the terms linear in w, the w-dependent component of weight modification in the NMDAR-based model is obtained,

$$\begin{aligned}\frac{d\langle w\rangle_w}{dt} &= \frac{A_+}{2}\,\bar{r}_{\rm post}\,\mu_w^{\rm up}\left(1+{\rm erf}\,(\frac{\mu^{\rm up}-\theta^{\rm up}}{\sqrt{2}\,\sigma_{\rm up}})\right)\\ &\quad-\frac{A_-}{2}\,r_{\rm pre}\,\mu_w^{\rm dn}\left(1+{\rm erf}\,(\frac{\mu^{\rm dn}-\theta^{\rm dn}}{\sqrt{2}\,\sigma_{\rm dn}})\right)\,.\end{aligned} \tag{S18}$$

.

Equation (S13) together with equation (S18) describe modification of the mean weight through NMDAR-base model,

$$\frac{d\langle w\rangle}{dt} = \frac{d\langle w\rangle_0}{dt} + \frac{d\langle w\rangle_{\langle w\rangle}}{dt}\,,$$

which is used in analytical results in figure 8. For small deviations of individual synaptic weights around the mean, equation (S18) can be used giving

$$\frac{d\,\delta w}{dt} = \frac{d\langle w\rangle_{\delta w}}{dt}\,,$$

which is used in figure 8B.
