# Supplementary material for: Stability and Competition in Multi-spike Models of Spike-Timing Dependent Plasticity
Source: PLoS Comput Biol. 2016 Mar 3;12(3):e1004750. doi: 10.1371/journal.pcbi.1004750 (PMC4777380; doi:10.1371/journal.pcbi.1004750)
Supplement: S1 Fig — (PDF) [file pcbi.1004750.s004.pdf]

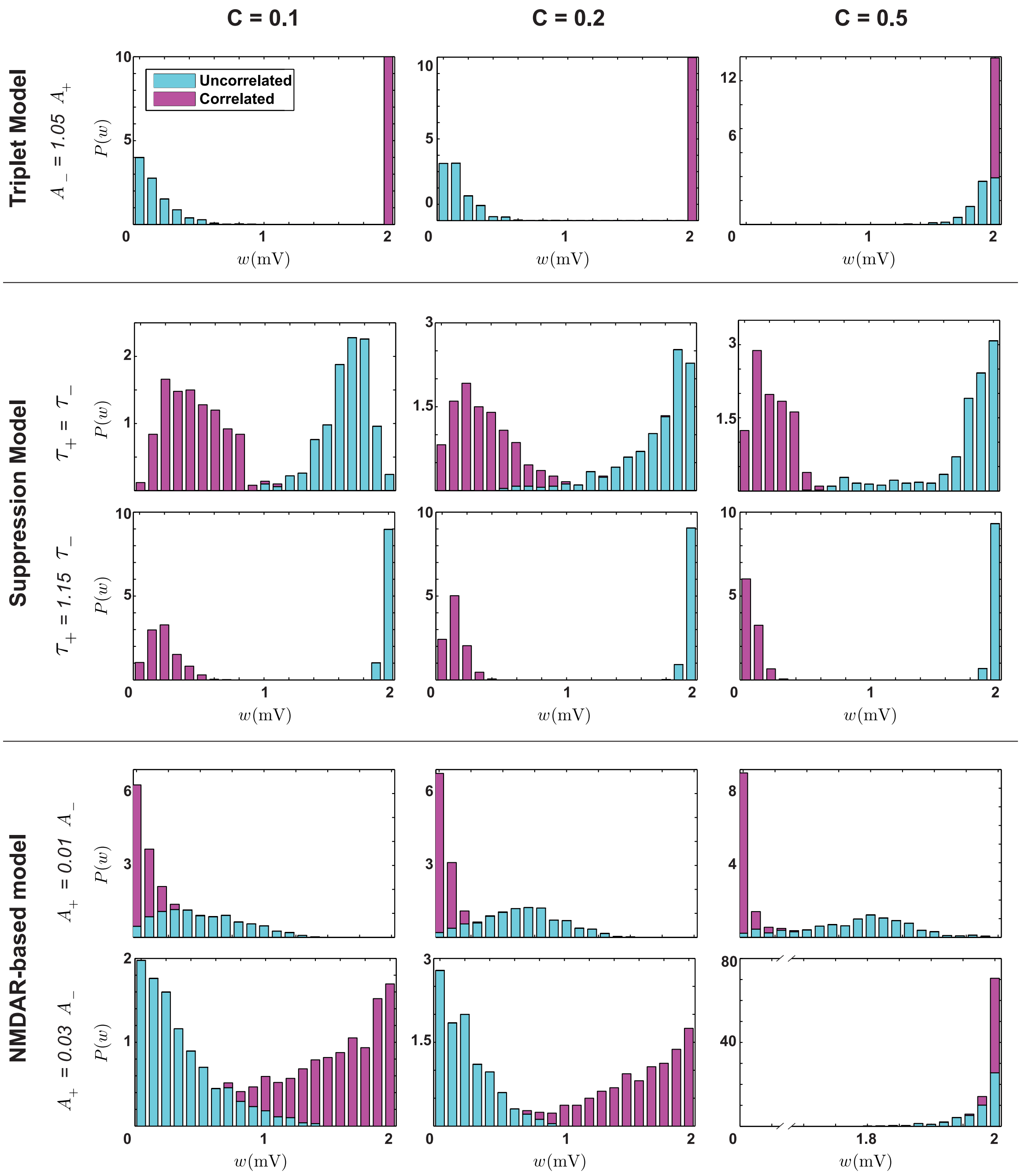

Figure 1: **S1 Figure. Synaptic competition with different levels of correlation.** The left, middle and right columns show synaptic competition with correlation coefficients of $c = 0.1$, $c = 0.2$ and $c = 0.5$, respectively. Each row corresponds to the results of different models in different parameter regimes. Note that the middle column shows the same results reported in the main text, but are repeated here for ease of comparison. In all models, the parameter regimes that were not competitive with $c = 0.2$, remain uncompetitive even with different values of c, so they are not shown. The already restricted competitive behavior of triplet model is lost when $c = 0.5$ (top row, right). Similarly, the Hebbian competition in NMDAR-based model disappears with $c = 0.5$ (bottom row, right). In all other cases, the competitive behavior of the models does not change qualitatively with different correlation coefficients.
